# 14-3-3 proteins regulate cullin 7-mediated Eag1 degradation

**DOI:** 10.1186/s13578-023-00969-w

**Published:** 2023-01-30

**Authors:** Chang-Heng Hsieh, Chia-Cheng Chou, Ya-Ching Fang, Po-Hao Hsu, Yi-Hung Chiu, Chi-Sheng Yang, Guey-Mei Jow, Chih-Yung Tang, Chung-Jiuan Jeng

**Affiliations:** 1 Institute of Anatomy and Cell Biology, College of Medicine, National Yang Ming Chiao Tung University, Taipei, 112 Taiwan; 2 National Laboratory Animal Center, National Applied Research Laboratories, Taipei, Taiwan; 3 Department of Physiology, College of Medicine, National Taiwan University, Taipei, 100 Taiwan; 4 School of Medicine, Fu-Jen Catholic University, New Taipei City, Taiwan; 5 Brain Research Center, National Yang Ming Chiao Tung University, Taipei, Taiwan

**Keywords:** Ubiquitin ligase, Protein stability, Potassium channel, Proteasomal degradation, Lysosomal degradation, Protein interaction

## Abstract

**Background:**

Mutations in the human gene encoding the neuron-specific Eag1 (K_V_10.1; KCNH1) potassium channel are linked to congenital neurodevelopmental diseases. Disease-causing mutant Eag1 channels manifest aberrant gating function and defective protein homeostasis. Both the E3 ubiquitin ligase cullin 7 (Cul7) and the small acid protein 14-3-3 serve as binding partners of Eag1. Cul7 mediates proteasomal and lysosomal degradation of Eag1 protein, whereas over-expression of 14-3-3 notably reduces Eag1 channel activity. It remains unclear whether 14-3-3 may also contribute to Eag1 protein homeostasis.

**Results:**

In human cell line and native rat neurons, disruptions of endogenous 14-3-3 function with the peptide inhibitor difopein or specific RNA interference up-regulated Eag1 protein level in a transcription-independent manner. Difopein hindered Eag1 protein ubiquitination at the endoplasmic reticulum and the plasma membrane, effectively promoting the stability of both immature and mature Eag1 proteins. Suppression of endogenous 14-3-3 function also reduced excitotoxicity-associated Eag1 degradation in neurons. Difopein diminished Cul7-mediated Eag1 degradation, and Cul7 knock-down abolished the effect of difopein on Eag1. Inhibition of endogenous 14-3-3 function substantially perturbed the interaction of Eag1 with Cul7. Further structural analyses suggested that the intracellular Per-Arnt-Sim (PAS) domain and cyclic nucleotide-binding homology domain (CNBHD) of Eag1 are essential for the regulatory effect of 14-3-3 proteins. Significantly, suppression of endogenous 14-3-3 function reduced Cul7-mediated degradation of disease-associated Eag1 mutant proteins.

**Conclusion:**

Overall these results highlight a chaperone-like role of endogenous 14-3-3 proteins in regulating Eag1 protein homeostasis, as well as a therapeutic potential of 14-3-3 modulators in correcting defective protein expression of disease-causing Eag1 mutants.

**Supplementary Information:**

The online version contains supplementary material available at 10.1186/s13578-023-00969-w.

## Background

The neuron-specific voltage-dependent ether-à-go-go (Eag) potassium (K^+^) (K_V_10) channel, a subtype of the KCNH channel family, is widely expressed in various regions of the mammalian brain, and may play a critical role in determining membrane excitability in dendrosomatic regions and axon terminals [1–6]. Mutations in the human *KCNH1* gene encoding the Eag1 (K_V_10.1; KCNH1) protein, an isoform of the Eag K^+^ channel, have been linked to two congenital neurodevelopmental diseases, the Temple-Baraitser syndrome (TMBTS) and the Zimmermann-Laband syndrome (ZLS), both of which are characterized by intellectual disability, facial dysmorphism, and hypoplasia or aplasia of nails and terminal phalanges [7–9]. Disease-causing mutant Eag1 channels are associated with aberrant voltage-dependent gating function, as well as defective protein homeostasis (proteostasis) [8–11].

The molecular mechanisms governing protein homeostasis of the Eag1 K^+^ channel remains elusive. By performing yeast two-hybrid screening of a rat brain library, we previously identified two novel binding partners of Eag1, cullin 7 (Cul7) and makorin ring finger protein 1 (MKRN1; also known as RNF61), that differentially regulate Eag1 protein degradation [10, 11]. Cul7, serving as a scaffold protein, assembles with the adaptor protein Skp1, the substrate-targeting subunit Fbw8, and the really interesting (RING)-domain protein ROC1 (Rbx1) to function as a subtype of the cullin-based E3 ubiquitin ligase [12, 13]. Biochemical and morphological evidence supports the notion that Cul7 promotes proteasomal and lysosomal degradation of Eag1 proteins localized at the endoplasmic reticulum (ER) and the plasma membrane, respectively [11]. In contrast, MKRN1 is a monomeric E3 ubiquitin ligase that contains a RING-finger E2-binding domain and a substrate-binding domain in the same molecule [14, 15]. Unlike the proteostatic role of Cul7 in mediating both ER and plasma membrane quality control of Eag1, MKRN1 appears to exclusively promote polyubiquitination and proteasomal degradation of ER-localized immature Eag1 protein [10]. Significantly, both Cul7 and MKRN1 contribute to degradation of disease-causing mifolded Eag1 mutant proteins [10, 11].

14-3-3 proteins, ubiquitously expressed in different tissues including the brain, comprise seven isoforms in mammals and function as homo- or hetero-dimers [16–18]. Through either phosphorylation-dependent or -independent modulation of the protein conformation of their substrates in the brain, 14-3-3 proteins modulate a wide variety of different neuronal signaling processes, such as neurotransmission, synaptic plasticity, brain development, and neurodegenerative diseases [19–22]. Interestingly, we demonstrated previously that 14-3-3 proteins serve as Eag1 binding partners, and that over-expression of the 14-3-3θ isoform notably reduces Eag1 K^+^ channel activity via a phosphorylation-independent mechanism [23].

Emerging evidence indicates that 14-3-3 proteins also play chaperone-like roles by regulating proteostasis of their substrate proteins [24–27]. It remains unclear, however, whether 14-3-3 proteins may take part in the homeostatic mechanisms of Eag1 protein. To address this important question, in this study we aim to investigate the contribution of endogenous 14-3-3 proteins in Eag1 proteostasis in both human cells and rat neurons. Multiple lines of biochemical, cell biological, and pharmacological evidence support the notion that 14-3-3 proteins facilitate Eag1 degradation mediated by Cul7, but not MKRN1. Moreover, suppression of endogenous 14-3-3 function considerably rescues defective protein expression of mutant Eag1 proteins associated with TMBTS and ZLS. Our findings demonstrate a chaperone-like role of 14-3-3 in Cul7-mediated Eag1 protein degradation.

## Results

### Suppression of endogenous 14-3-3 promotes Eag1 protein level

**Fig. 1:**
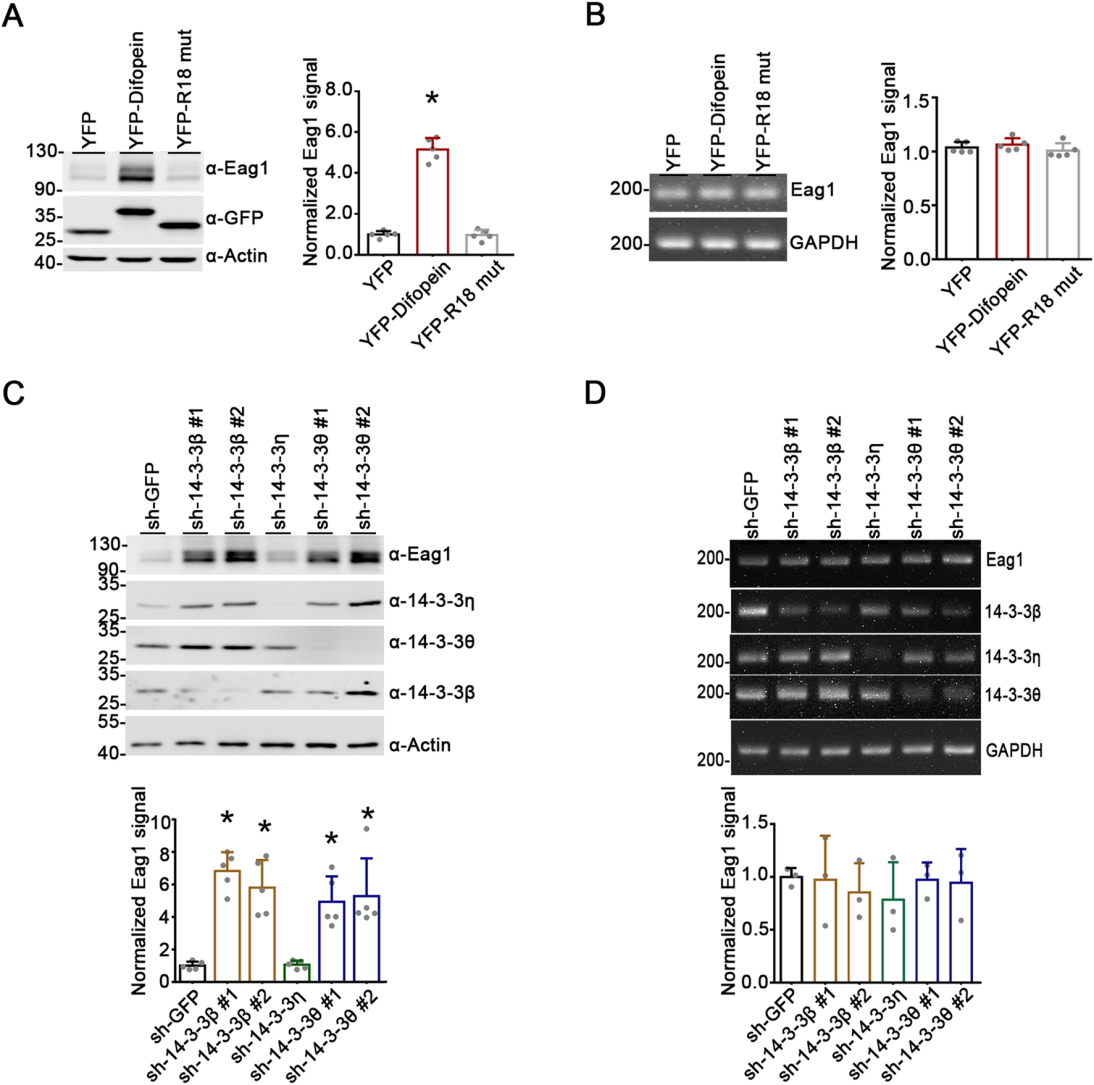
Difopein increases Eag1 protein expression. **A** (*Left*) Representative immunoblot showing the effect of difopein co-expression on Eag1 over-expressed in HEK293T cells. Cells were transfected with cDNAs for Eag1, as well as YFP vector, YFP-difopein, or YFP-R18 mutant (YFP-R18 mut). Two days post-transfection, cells were subject to immunoblotting analyses with the anti-Eag1 (α-Eag1), anti-GFP (α-GFP), and anti-β-actin (α-actin) antibodies. (*Right*) Quantitative analyses of relative Eag1 protein levels for the three co-transfection conditions. Protein densities were standardized as the ratio to the cognate β-actin signals, followed by normalization with respect to the YFP vector control (*, P < 0.05; n = 5). **B** Lack of effect of difopein co-expression on Eag1 mRNA level in HEK293T cells. Semi-quantitative RT-PCR analyses of relative Eag1 mRNA levels were employed for the three co-expression conditions. mRNA levels of Eag1 were standardized as the ratio of Eag1 signals to the cognate GAPDH mRNA levels, followed by normalization with respect to the YFP vector control (n = 3). **C–D** Effects of shRNA knockdown of various endogenous 14-3-3 proteins on Eag1 protein (**C**) or mRNA (**D**) levels in HEK293T cells. HEK293T cells over-expressing Eag1 were subject to infection with a control shRNA for GFP (sh-GFP), or shRNA specific for 14-3-3β, η, or θ isoforms (sh-14-3-3β#1, sh-14-3-3β#2, sh-14-3-3η, sh-14-3-3θ#1, sh-14-3-3θ#2). Quantitative analyses of relative Eag1 protein levels are based on normalization with respect to the sh-GFP control (*, P < 0.05; n = 4). Lack of effect of 14-3-3 knockdown on Eag1 mRNA levels is supported by quantitative analyses of relative Eag1 mRNA levels normalized with respect to the sh-GFP control (n = 5)

We began by asking whether endogenous 14-3-3 proteins may regulate protein homeostasis of Eag1 K^+^ channels over-expressed in HEK293T cells. To this end, the interaction between endogenous 14-3-3 proteins and their ligands were effectively disrupted by over-expressing difopein (dimeric fourteen-three-three peptide inhibitor), a high-affinity 14-3-3 inhibitor that comprises two R18 (known for its 14-3-3-binding specificity) peptides connected by a linker and competitively binds to all 14-3-3 isoforms in a phosphorylation-independent manner [28]. Furthermore, we also examined the effect of over-expressing an inactive mutant control of difopein, the monomeric R18 mutant, wherein two key acidic residues essential for 14-3-3 binding in R18 have been replaced with lysines, thereby preventing the R18 mutant from interacting with Eag1 (Additional file 1: Fig. S1) [28]. Figure 1A demonstrates that co-expression with YFP-tagged difopein led to almost five-fold increase in Eag1 protein level in HEK293T cells. In comparison, no discernible effect was observed when we co-expressed Eag1 with YFP-tagged R18 mutant. This protein up-regulation effect did not appear to result from difopein-induced change in Eag1 transcription, as semi-quantitative RT-PCR analyses indicated a lack of effect of difopein on Eag1 mRNA level (Fig. 1B).

In addition to the pan 14-3-3 inhibitor difopein, we also employed the RNA interference technique to suppress specific endogenous 14-3-3 isoforms in HEK293T cells. Among the seven 14-3-3 isoforms, Eag1 displays significantly higher binding efficiency to β, η, and θ [23]. Upon shRNA knock-down of endogenous 14-3-3 isoforms β or θ, but not η, we observed a sizeable up-regulation of total Eag1 protein level in HEK293T cells (Fig. 1C), despite the absence of a detectable change in the mRNA level of Eag1 (Fig. 1D). Together, these results imply that disruption of endogenous 14-3-3 function may promote Eag1 protein level via a transcription-independent mechanism.

### Endogenous 14-3-3 modulates Eag1 protein stability

Unlike the significant proteostatic effect of endogenous 14-3-3 proteins, we previously reported that co-expression with the 14–3-3θ isoform decreased Eag1 K^+^ channel activity without notably affecting Eag1 protein level or membrane trafficking [23]. Rather, our functional analyses suggested that 14-3-3-3θ over-expression rendered a fraction of the plasma membrane-localized Eag1 channel locked in a non-conducting state [23]. In line with the foregoing observations, over-expression of the 14-3-3 isoforms β, ε, γ, η, θ, or ξ failed to substantially alter Eag1 mRNA and/or protein levels (Additional file 1: Fig. S2). This discrepancy between over-expressed and endogenous 14-3-3 on Eag1 protein homeostasis raises the possibility that, while over-expressed 14-3-3 proteins preferentially modulate cell-surface Eag1 channel activity, endogenous 14-3-3 proteins mainly control Eag1 proteostatic processes. To address this hypothesis, we went on to test the idea that endogenous 14–3-3 may regulate Eag1 protein stability.

**Fig. 2:**
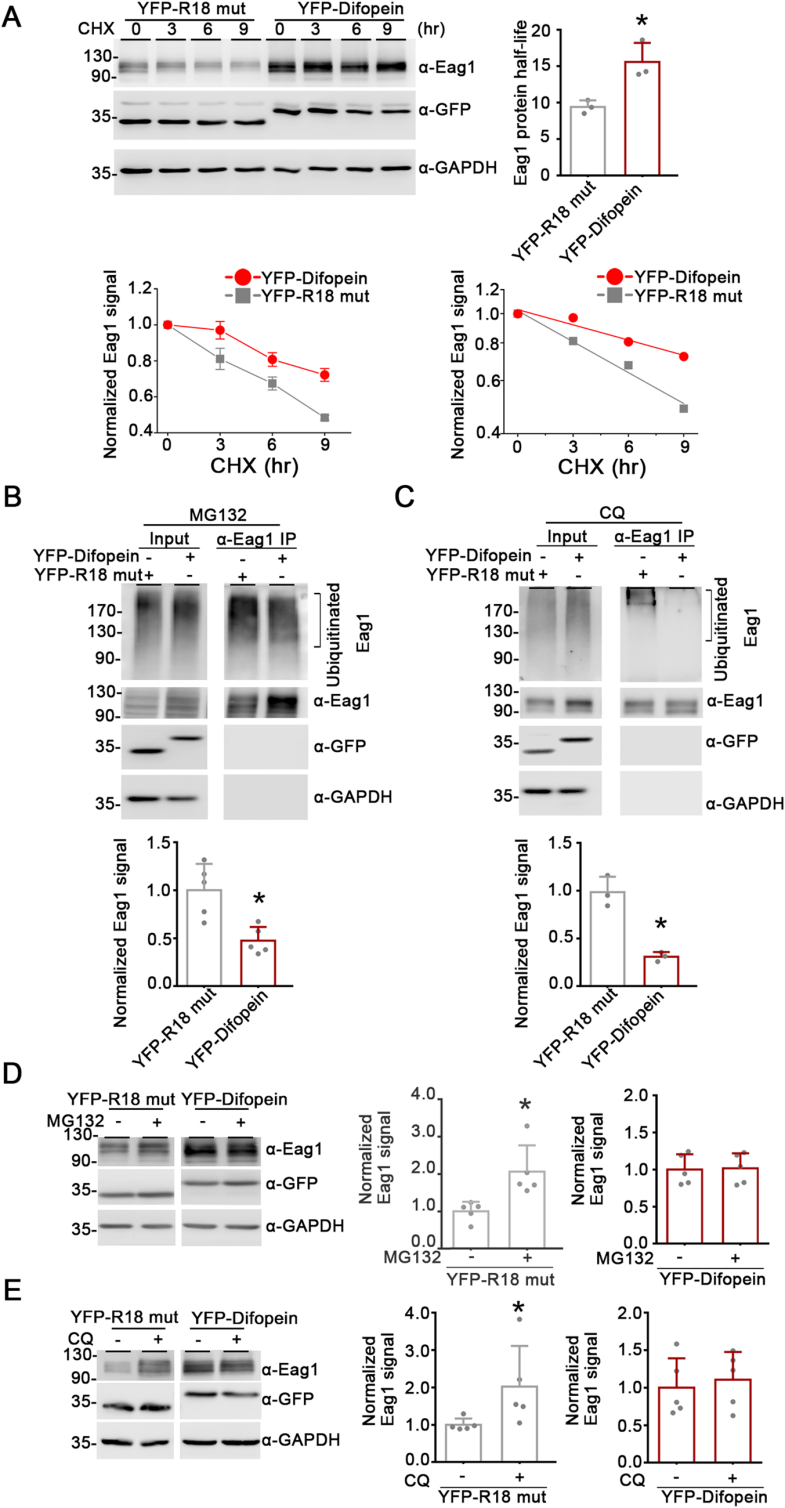
Difopein promotes Eag1 protein stability. **A** Difopein co-expression enhances Eag1 protein half-life values in HEK293T cells. (*Upper left*) Representative immunoblots showing the protein turn-over time course of Eag1 co-expressed with R18 mutant or difopein in HEK293T cells. 48 h post-transfection, cells were subject to 100 μg/ml cycloheximide (CHX) treatment for the indicated durations. (*Lower left*) Linear plot of relative Eag1 protein levels in response to different CHX treatment durations. Protein densities were standardized as the ratio of Eag1 signals to the cognate GAPDH signals, followed by normalization with respect to the no-CHX-treatment (0 h) control. (*Lower right*) Semi-logarithmic plot of linear-regression analyses (*solid lines*) of the same data points shown to the left. (*Upper right*) Statistical comparisons of Eag1 protein half-life values for the two co-expression conditions (*, P < 0.05; n = 3). **B**, **C** Difopein co-expression diminishes Eag1 protein ubiquitination. Transfected cells were treated with 10 μM MG132 (**B**) or 100 μM chloroquine (CQ) (**C**) for eight hours, followed by immunoprecipitation (IP) with the anti-Eag1 antibody. (*Top panels*) Representative immunoblot showing Eag1 ubiquitination in the absence or presence of difopein co-expression. Ubiquitinated Eag1 is visualized as high-molecular-weight protein smears detected with the FK2 anti-ubiquitin antibody. (*Bottom panels*) Quantification of relative ubiquitinated Eag1 levels normalized with respect to the R18 mutant control (*, P < 0.05; n = 3). **D**, **E** Difopein co-expression abolishes MG132- (**D**) and CQ- (**E**) induced increase in Eag1 protein level. (*Left panels*) Representative immunoblots. (*Right panels*) Quantitative analyses of the effect of MG132 (**D**) and CQ (**E**) treatments on Eag1 protein levels for the two co-expression conditions. Protein densities were standardized as the ratio to the cognate GAPDH signals, followed by normalization with respect to the corresponding vehicle-treated control (*, P < 0.05; n = 6)

As revealed by the cycloheximide (CHX) chase assay shown in Fig. 2A, suppression of endogenous 14-3-3 protein function with difopein dramatically enhanced Eag1 protein half-life by more than 65%, from about 9.4 h to about 15.6 h. Eag1 is subject to ubiquitin (Ub)-mediated proteasomal and lysosomal degradation [11]. Importantly, Eag1 polyubiquitination, manifesting as diffuse Ub-conjugated Eag1 protein smear in the presence of the proteasome inhibitor MG132 or the lysosome inhibitor chloroquine (CQ), was substantially attenuated upon difopein co-expression (Fig. 2B, C). Moreover, the up-regulation effect of MG132 and CQ treatment on Eag1 protein level was efficiently abolished by difopein, but not its inactive mutant control R18 (Fig. 2D, E). Taken as a whole, these data suggest that difopein co-expression may hinder key Eag1 ubiquitination process prior to its degradation by the proteasome and lysosome. In other words, endogenous 14-3-3 may promote Eag1 protein ubiquitination, thereby enhancing proteasomal and lysosomal degradation of the K^+^ channel.

**Fig. 3:**
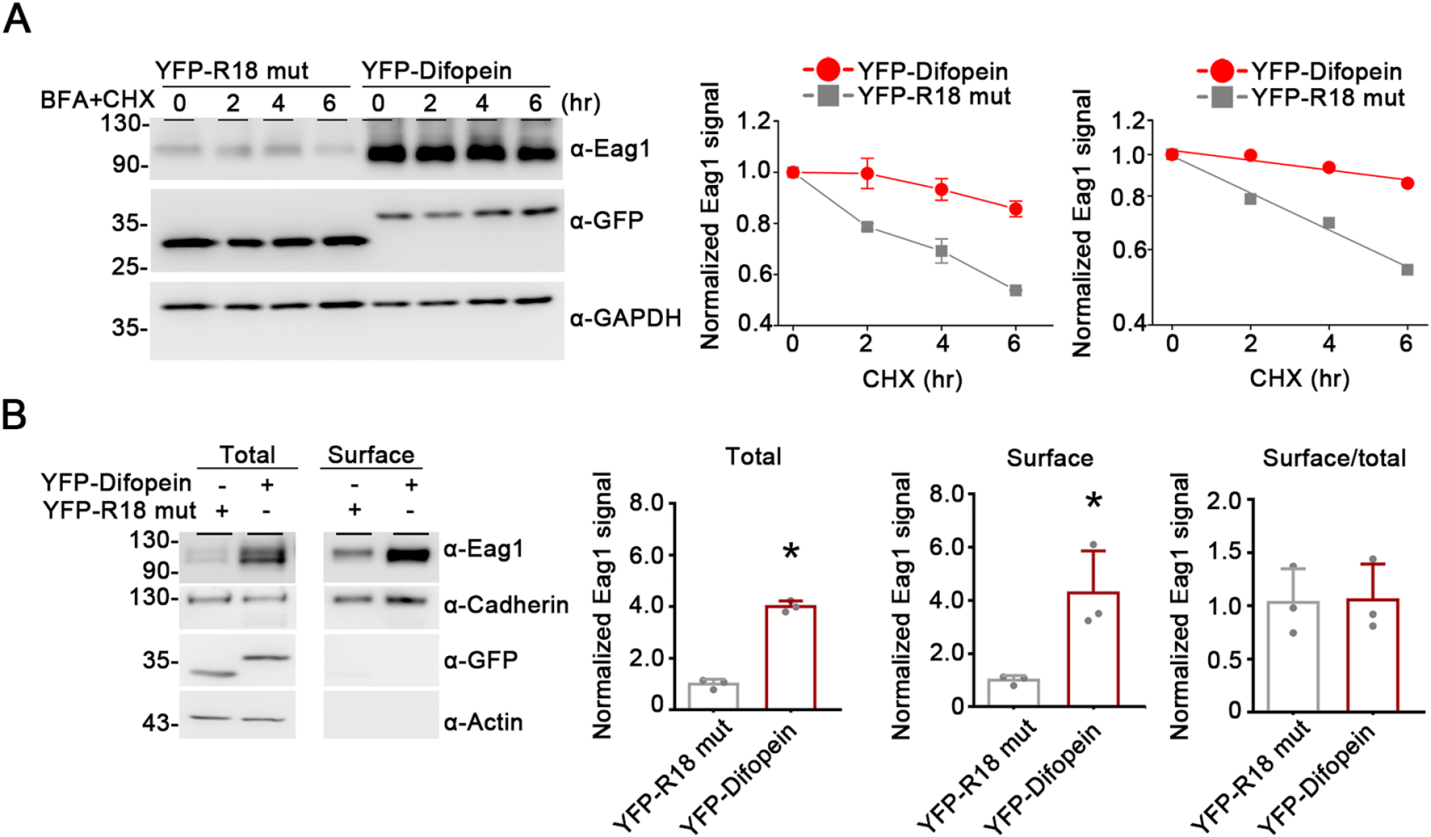
Difopein increases both immature and mature Eag1 protein levels. **A** Difopein co-expression promotes immature Eag1 protein stability in HEK293T cells. *(Left*) Representative immunoblot showing the effect of R18 mutant or difopein co-expression on Eag1 protein turn-over time course in the presence of brefeldin A (BFA). Transfected HEK293T cells were pretreated with BFA (10 μM) for 12 h, followed by cycloheximide (CHX) treatment for the indicated duration. (*Center*) Linear plot of Eag1 protein degradation time course in the presence of BFA treatment. (*Right*) Semi-logarithmic plot of linear-regression analyses (*solid lines*) of the same data points shown to the left. Protein densities were standardized as the ratio of Eag1 signals to the cognate GAPDH signals, followed by normalization with respect to the corresponding no-CHX-treatment (0 h) control. Data points represent the average of four independent experiments. **B** Difopein co-expression augments cell-surface Eag1 protein level. (*Left panels*) Representative immunoblots. Cell lysates from biotinylated intact cells were either directly employed for immunoblotting analyses (*Total*) or subject to streptavidin pull-down prior to immunoblotting analyses (*Surface*). Actin was used as the loading control. (*Right panels*) Quantification of total and surface protein levels, as well as membrane trafficking efficiency (Surface/total). Total and surface protein densities were standardized as the ratio to the cognate total actin signal, followed by normalization with that of the R18 mutant control. Membrane trafficking efficiency was calculated as surface protein density divided by the corresponding standardized total protein density, followed by normalization with respect to the surface/total ratio of the R18 mutant control (*, P < 0.05; n = 3)

Like other membrane proteins, biogenesis of Eag1 K^+^ channels begins at the ER, followed by further protein maturation processes at the Golgi and the ensuing membrane trafficking to the cell surface. This progression of Eag1 biogenesis from immature to mature proteins manifests differential glycosylation patterns [10, 11]. To further investigate the role of endogenous 14-3-3 proteins in Eag1 proteostasis, we then focused on the effect of difopein on immature and mature Eag1 proteins. In the presence of prolonged incubation with brefeldin A (BFA), which blocks forward trafficking of proteins from the ER to the Golgi [29], the vast majority of Eag1 K^+^ channels in HEK293T cells would be trapped in the ER and therefore remain in the immature form. As illustrated in Fig. 3A, CHX chase assays in the presence of BFA indicated that co-expression with difopein resulted in a substantial increase of immature Eag1 protein half-life from about 9 h to almost 33 h. Furthermore, we performed surface biotinylation analyses to examine mature Eag1 protein level at the plasma membrane. Figure 3B highlights that difopein significantly promoted surface Eag1 protein expression by four-fold. Given its comparable effect on surface and total Eag1 protein levels (Fig. 3B), difopein did not seem to detectably affect Eag1 membrane trafficking. Therefore, the observed increase in Eag1 surface expression very likely reflects an enhanced stability of mature Eag1 protein in the presence of difopein.

**Fig. 4:**
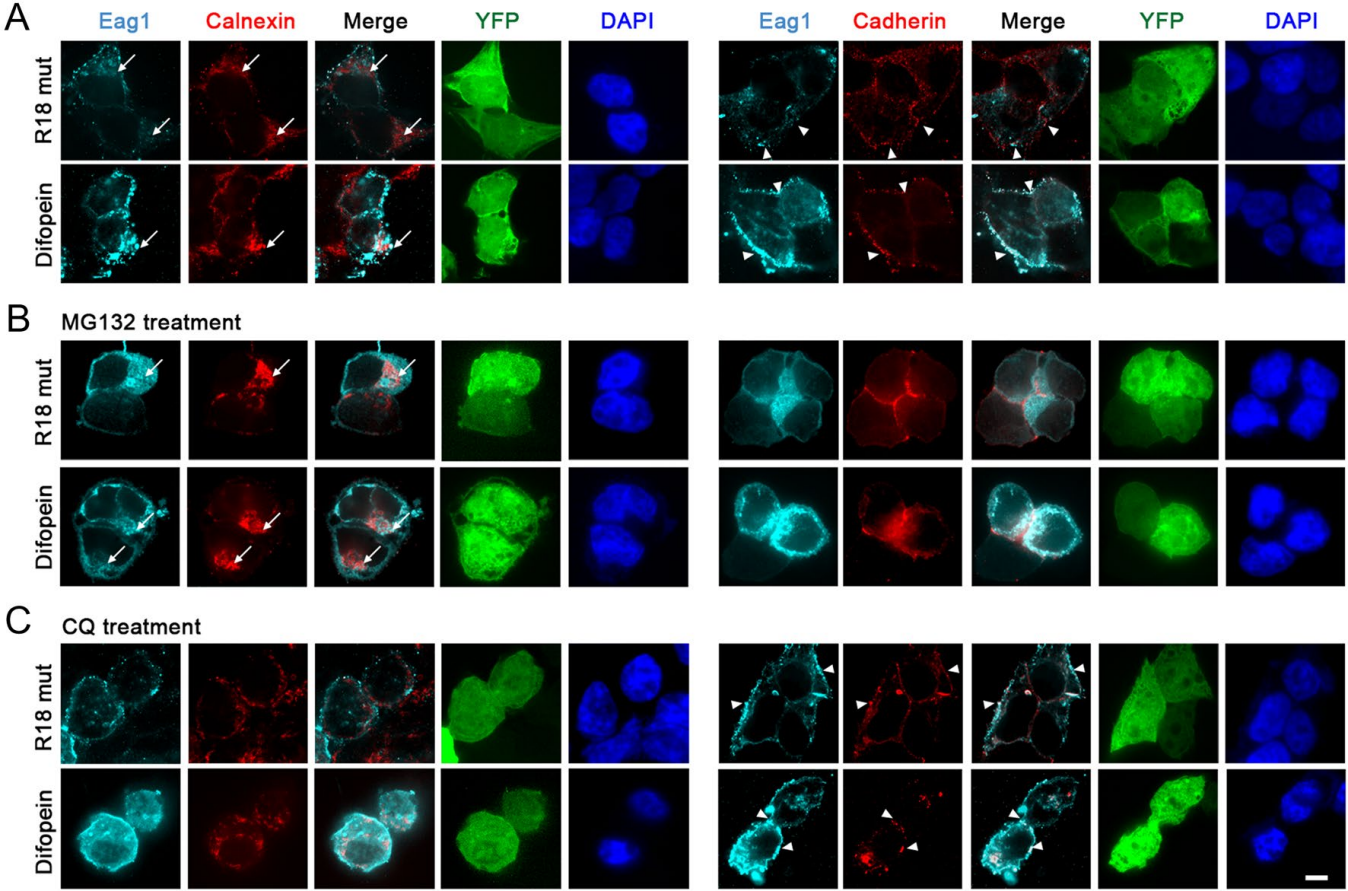
Difopein enhances both ER and cell-surface expression of Eag1. Representative confocal micrographs showing the effect of R18 mutant and difopein co-expression on Eag1 immunofluorescent signals in HEK293T cells, in the absence (**A**) or presence of 12-h treatment with 10 μM MG132 (**B**) or 100 μM chloroquine (CQ) (**C**). Eag1 was detected with the anti-Eag1 antibody (*cyan*), nuclei were counterstained with DAPI (*blue*), and YFP-tagged proteins were directly visualized (*green*). ER and cell-surface localizations of Eag1 were determined by co-localization with the ER marker calnexin *(red; left panels)* and the plasma membrane marker cadherin (*red; right panels*), respectively. Merged images are shown in the third column of each panel. Arrows indicate intracellular ER staining, whereas arrowheads denote plasma membrane staining. Scale bar, 10 μm. Data shown here are representative of over 80 cells from at least three independent experiments. See Additional file 1: Fig. S3 for further quantitative analyses

By employing the confocal laser scanning microscopy, we also studied the effect of difopein on the subcellular localization of Eag1 over-expressed in HEK293T cells. The immunofluorescent images in Fig. 4A illustrate that the ER-resident protein calnexin co-localized with the presumably immature fraction of Eag1 proteins, whereas the plasma membrane protein cadherin was detected along with the presumably mature Eag1. Co-expression with difopein promoted Eag1 protein signals at both the ER and the plasma membrane (Fig. 4A–C) (Additional file 1: Fig. S3). In contrast, treatment with the proteasome inhibitor MG132 and the lysosome inhibitor CQ preferentially enhanced perinuclear ER and cell-surface Eag1 staining, respectively (Fig. 4B, C) (Additional file 1: Fig. S3). Altogether, the aforementioned observations are consistent with the idea that suppression of endogenous 14-3-3 function with difopein may hinder Eag1 protein ubiquitination at the ER and the plasma membrane, thereby promoting the stability of both immature and mature Eag1 proteins.

### Regulation of neuronal Eag1 protein expression by endogenous 14-3-3

**Fig. 5:**
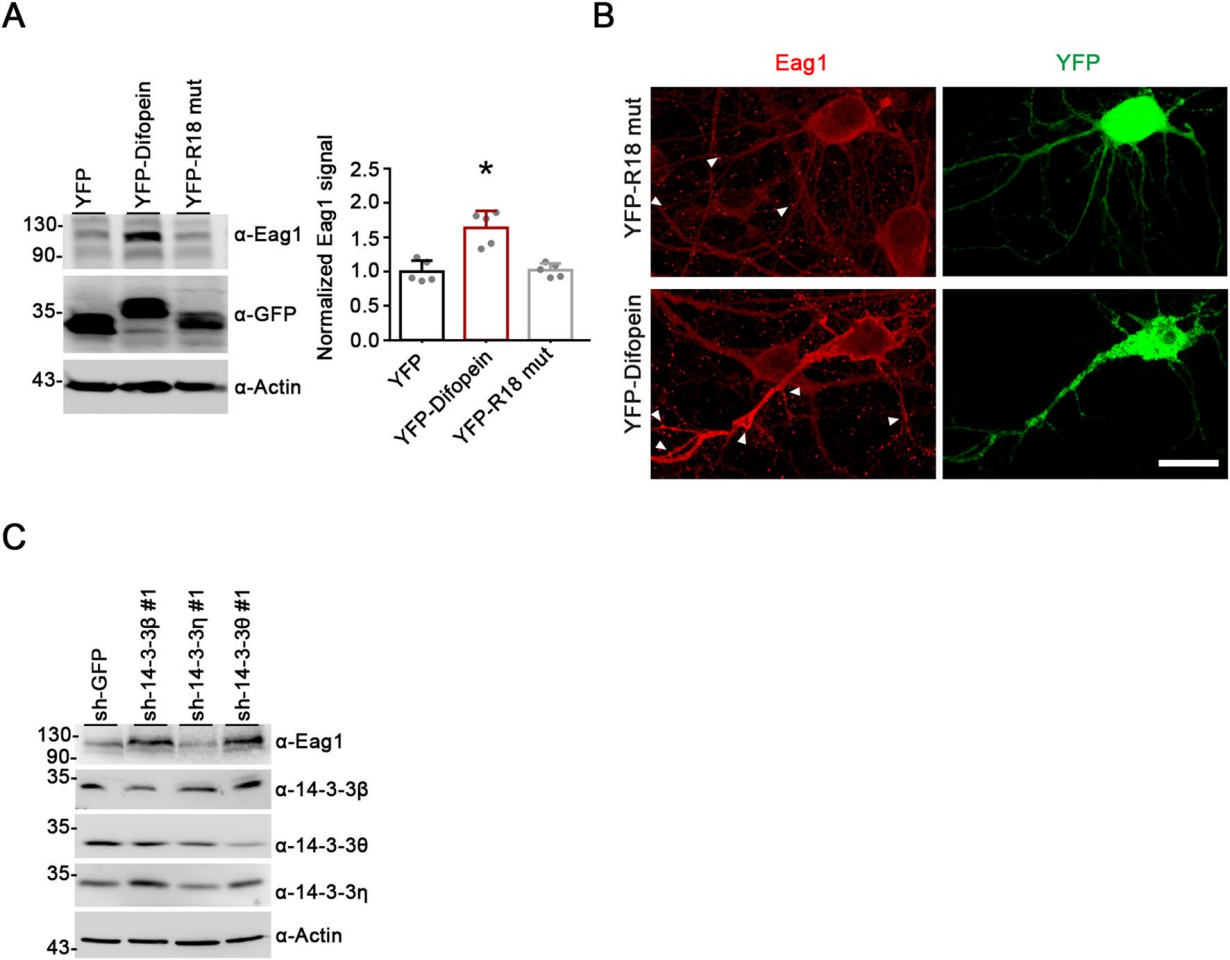
Difopein up-regulates Eag1 protein expression in neurons. **A**, **B** Over-expression of difopein promotes endogenous Eag1 expression in cultured cortical neurons. Neurons (DIV10) were transfected with YFP, YFP-difopein, or YFP-R18 mutant, and then incubated for two days, followed by immunoblotting (**A**) or immunofluorescent (**B**) analyses. Quantitative analyses of immunoblots are summarized by the bar graphs. Protein densities were standardized as the ratio to the cognate GAPDH signals, followed by normalization with respect to the YFP vector control (*, P < 0.05; n = 5). Endogenous Eag1 was detected with the anti-Eag1 antibody (*red*), and YFP-tagged proteins were directly visualized (*green*). Arrowheads denote punctate Eag1 staining patterns. Scale bar, 10 μm. See Additional file 1: Fig. S4 for further quantitative analyses of immunofluorescent images. **C** shRNA knockdown of 14-3-3β and 14-3-3θ, but not 14-3-3η, increases endogenous Eag1 protein level in cultured cortical neurons. Neurons (DIV10) were infected with various viral stocks and selected with puromycin; two days post-infection, immunoblotting analyses were performed using the indicated antibodies

Since Eag1 is a neuron-specific K^+^ channel abundantly expressed in the brain, next we examined whether difopein may also modulate endogenous Eag1 protein level in native neurons. Over-expression of difopein, but not the inactive R18 mutant, in cultured cortical neurons led to a sizable up-regulation of endogenous Eag1 protein expression by more than 50% (Fig. 5A). Similarly, immunofluorescent analyses showed that difopein notably increased Eag1 fluorescence signals in cultured hippocampal neurons (Fig. 5B) (Additional file 1: Fig. S4). Moreover, consistent with the foregoing finding in HEK293T cells (see Fig. 1C), shRNA knock-down of endogenous 14-3-3 isoforms β or θ, but not η, significantly enhanced endogenous Eag1 protein level in cultured cortical neurons (Fig. 5C). These data support the notion that suppression of 14-3-3 function/expression effectively up-regulates Eag1 protein expression in neurons.

**Fig. 6:**
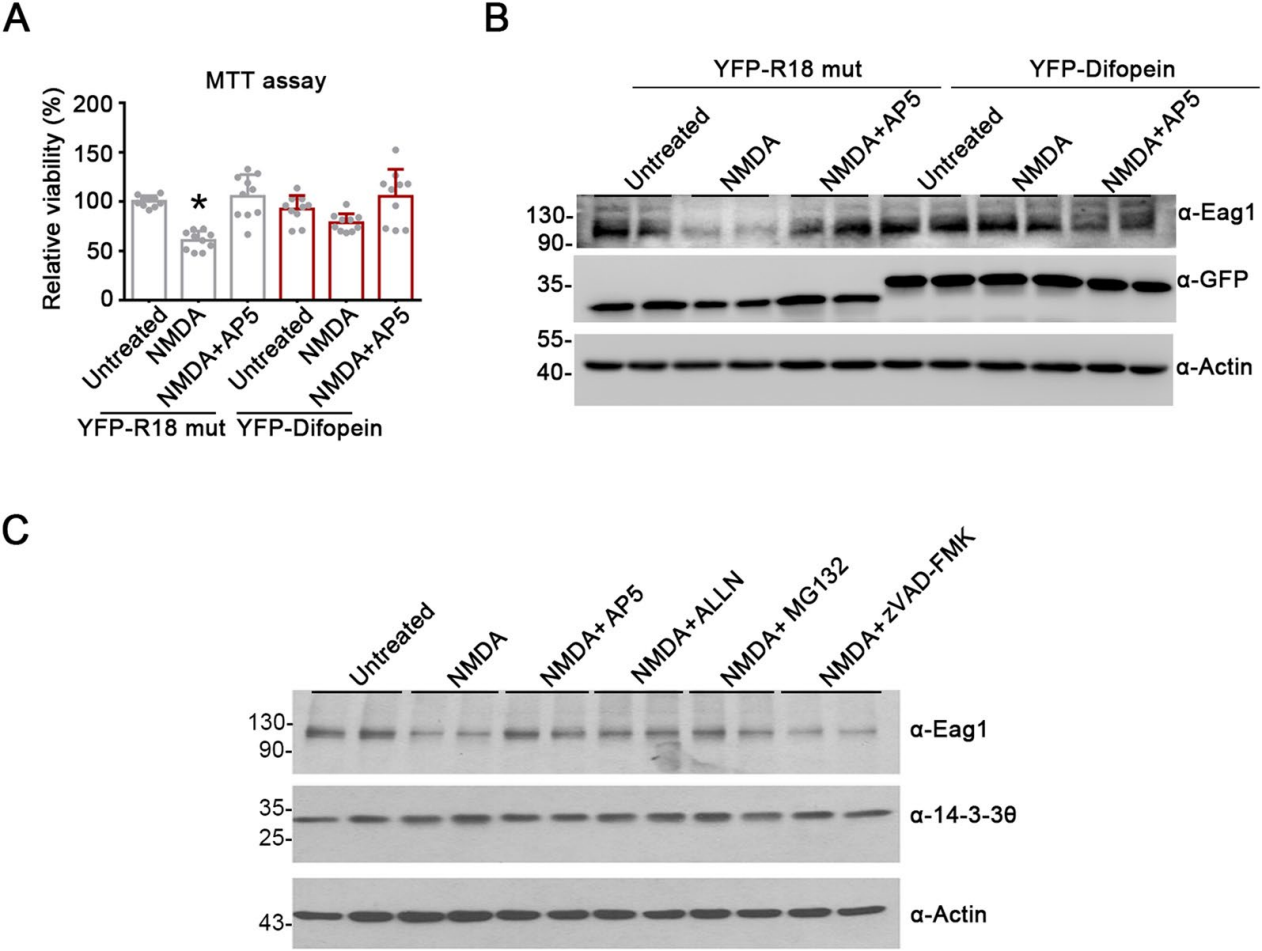
Effects of difopein expression on NMDA-induced neuronal excitotoxicity. **A**, **B** Difopein rescues NMDA excitotoxicity and averts NMDA-mediated reduction of endogenous Eag1 protein level in cultured cortical neurons. Neurons (DIV10) were subject to transfection with either R18 mutant or difopein. Two days post-transfection, neurons were stimulated with 20 μM NMDA for six hours, in the absence or presence of 30-min pretreatment with the NMDA receptor antagonist AP5 (50 μM), followed by MTT assays (**A**) or immunoblotting analyses with the indicated antibodies (**B**). Cell viability is expressed as the relative optical density at 540 nm of the mitochondria-produced formazan with respect to the non-NMDA-treatment (Untreated) control of YFP-R18-mut-transfected neurons. Statistical comparisons were performed with respect to the untreated group of R18 mutant-transfected neurons (*, P < 0.05; n = 9). **C** Proteasome inhibition prevents NMDA-mediated reduction of endogenous Eag1 protein level in cultured cortical neurons. Neurons (DIV12) were pretreated with the NMDA receptor antagonist AP5 (50 μM), the proteasomal inhibitors ALLN (10 μM) and MG132 (20 μM), or the caspase inhibitor zVAD-FMK (20 μM) for 30 min. Cells were then treated with 20 μM NMDA for 12 h in the presence of the specified inhibitors, followed by immunoblotting analyses with the indicated antibodies

**Fig. 7:**
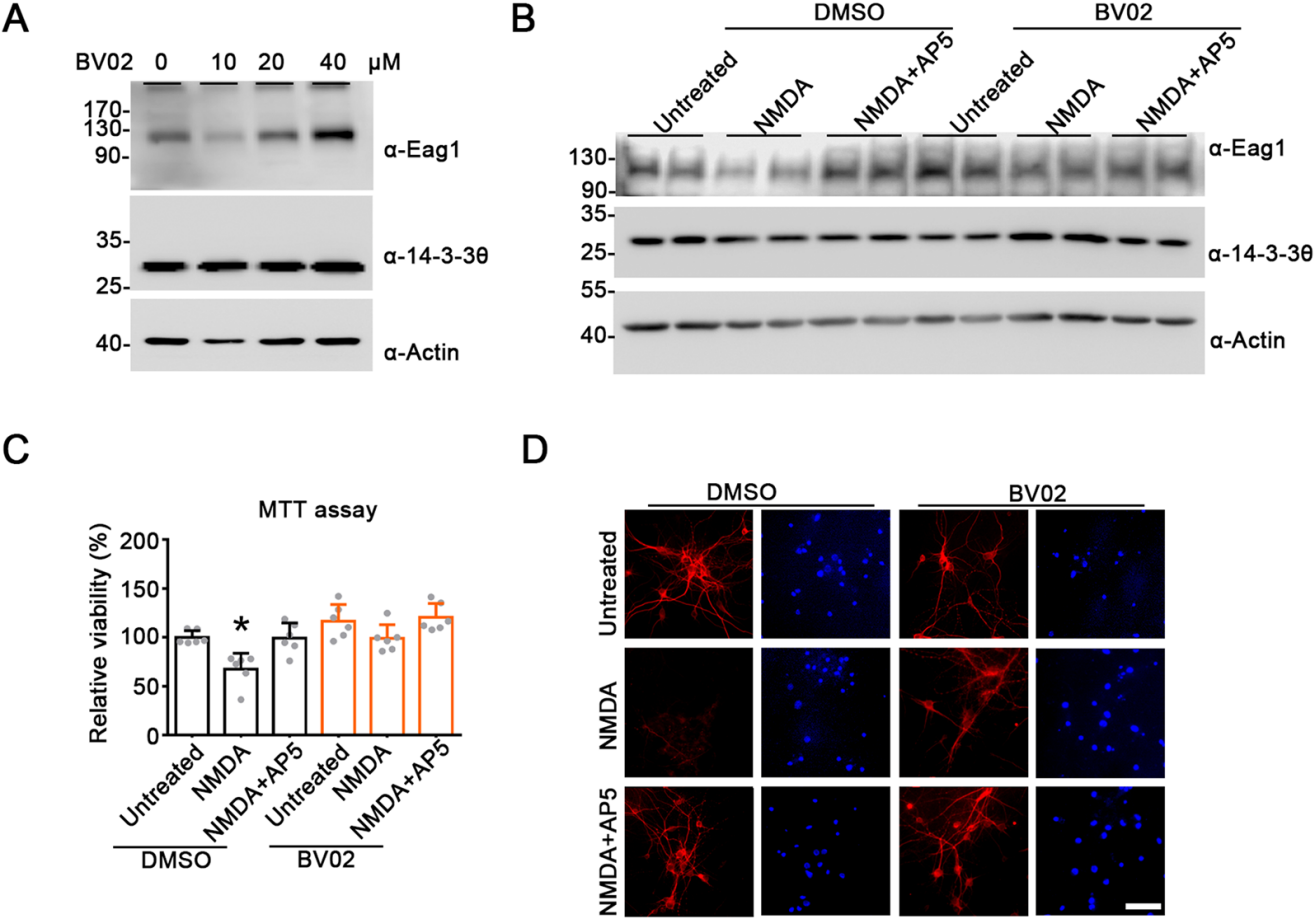
Effects of BV02 treatment on NMDA-induced neuronal excitotoxicity. **A** Treatment with the small-molecule 14-3-3 inhibitor BV02 (40 μM) promotes endogenous Eag1 protein level in cultured cortical neurons. Neurons (DIV12) were pretreated with the indicated concentrations of BV02 for six hours, followed by immunoblotting analyses with the indicated antibodies. **B**–**D** BV02 (40 μM) treatment averts NMDA-mediated reduction of endogenous Eag1 protein level and rescues NMDA excitotoxicity in cultured cortical neurons. Neurons were pretreated with DMSO or BV02, and then subject to 6-h treatment of 20 μM NMDA (in the absence or presence of 30-min 50-μM AP5 pretreatment), followed by immunoblotting analyses (**B**), MTT assay (**C**), or immunofluorescent inspections (**D**). Cell viability is expressed as the relative optical density at 540 nm of formazan with respect to the non-NMDA-treatment (Untreated) control of DMSO-treated neurons. Statistical comparisons were performed with respect to the untreated group of DMSO-treated neurons (*, P < 0.05; n = 5). For immunofluorescent experiments, neurons were stained with the anti-MAP2 antibody (*red*) and the nucleus counterstain DAPI (*blue*). NMDA treatment led to a significant diminishment of immunofluorescent signals of MAP2 (but not those of DAPI), which was prevented by pretreatment with AP5 or BV02. Scale bar, 10 μm

In response to excitotoxicity such as those caused by excessive activation of glutamatergic N-methyl-D-aspartate (NMDA) receptors [30, 31], viability of cultured cortical neurons substantially deteriorated and endogenous Eag1 protein level was dramatically reduced (Fig. 6A, B). This observation is consistent with previous reports that neuronal toxicity leads to K^+^ channel dysfunction, which may further disrupt cell survival in the brain [32–34]. Importantly, over-expression of difopein rescued NMDA excitotoxicity, as well as abolishing NMDA-induced Eag1 protein reduction (Fig. 6A, B). Furthermore, pretreatment with the proteasome inhibitors ALLN and MG132, but not the caspase inhibitor zVAD-FMK, averted excitotoxicity-induced Eag1 channel reduction (Fig. 6C), suggesting that NMDA excitotoxicity may promote proteasomal degradation of endogenous Eag1 K^+^ channel in neurons. In line with the effect of difopein over-expression, treatment of cultured cortical neurons with 40 μM BV02, a small-molecule 14-3-3 inhibitor [35], notably promoted neuronal Eag1 protein level (Fig. 7A). BV02 treatment also effectively abolished NMDA-induced Eag1 protein reduction (Fig. 7B), as well as alleviating NMDA excitotoxicity (Fig. 7C, D). Overall, these results suggest that preemptive inhibition of endogenous 14-3-3 proteins may effectively protect neurons from NMDA excitotoxicity, thereby preventing excitotoxicity-induced proteasomal degradation of Eag1 protein.

### 14–3-3 facilitates Eag1 degradation by Cul7

So far, we have provided multiple lines of evidence indicating that, in both HEK293T cells and neurons, suppression of endogenous 14-3-3 protein function with difopein or BV02 profoundly hampers proteasomal and lysosomal degradation of Eag1 K^+^ channels. As mentioned above in the Introduction section, the multi-subunit Cul7-Skp1-Fbw8-ROC1 E3 ubiquitin ligase complex is responsible for both proteasomal and lysosomal degradation of immature and mature Eag1 proteins, respectively [11]. In comparison, the monomeric MKRN1 E3 ubiquitin ligase exclusively promotes ER-associated degradation of immature Eag1 protein (Fang et al., 2021). Importantly, in both HEK293T cells and cultured cortical neurons, endogenous Cul7 co-exists in the same protein complex with 14-3-3θ and Eag1 (Additional file 1: Fig. S5). Therefore, one potential mechanism underlying the enhancement of Eag1 protein level by difopein/BV02 is that endogenous 14–3-3 proteins may contribute to Eag1 degradation by Cul7, which in turn is hindered by inhibition of 14-3-3 function with difopein/BV02. To address this intriguing hypothesis, we went on to examine whether difopein co-expression may affect Cul7-mediated degradation of Eag1 proteins.

**Fig. 8:**
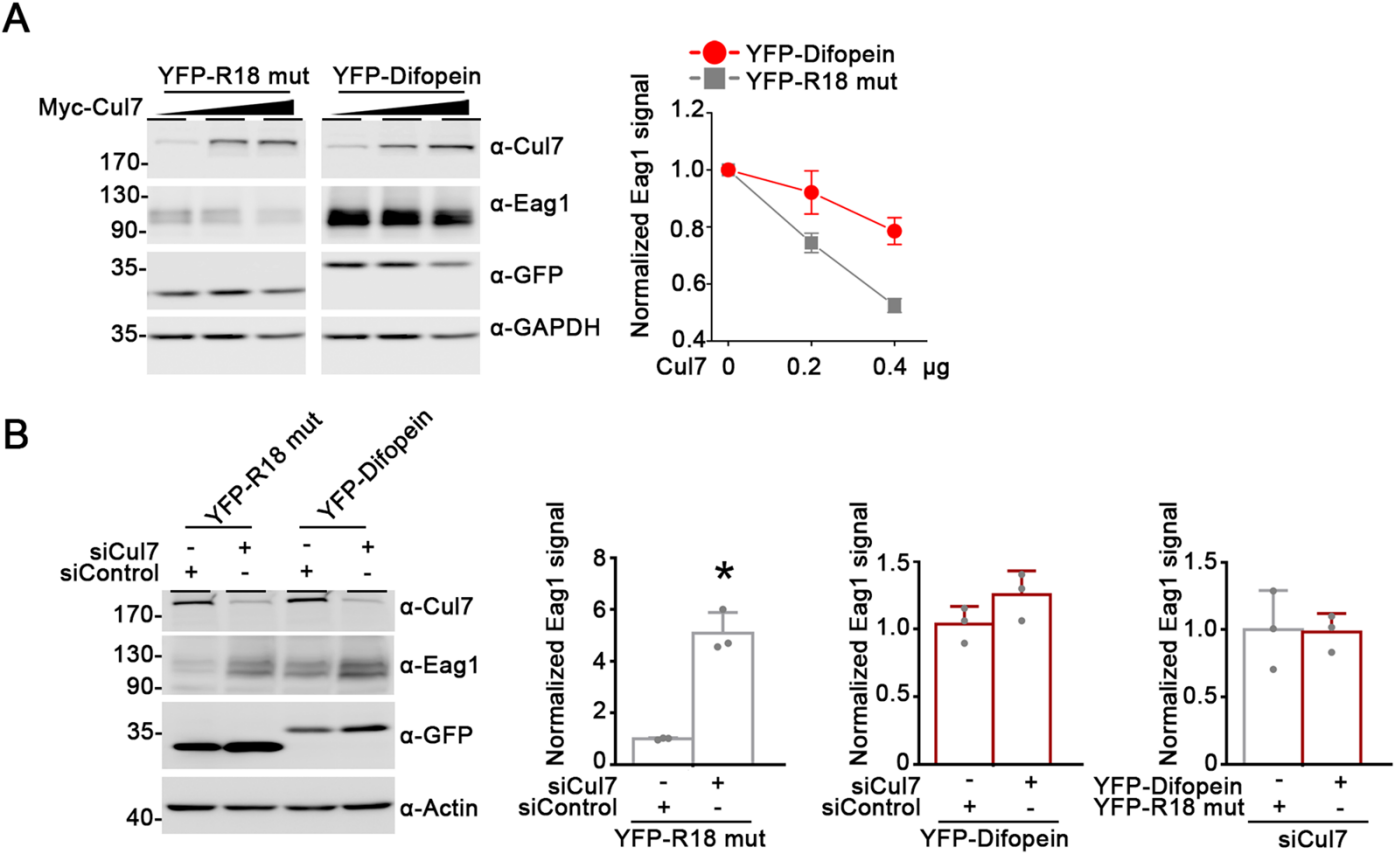
Difopein reduces Eag1 degradation by Cul7. **A** The effect of R18 mutant or difopein on Eag1 degradation by Myc-tagged Cul7 (Myc-Cul7) in HEK293T cells. *(Left*) Representative immunoblots. Eag1 was co-expressed with increasing amounts of Cul7. (*Right*) Quantification of relative Eag1 protein levels with respect to the amount of Cul7 used for co-transfection. Protein densities were standardized as the ratio of Eag1 signals to the cognate GAPDH signals, followed by normalization with respect to the Myc-vector control (n = 3). **B** The effect of siRNA knock-down of endogenous Cul7 (siCul7) on the regulation of Eag1 protein expression by R18 mutant or difopein in HEK293T cells. *(Left*) Representative immunoblots. (*Right*) Quantification of relative Eag1 protein levels. Protein densities were standardized as the ratio of Eag1 signals to the cognate β-actin signals, followed by normalization with respect to the corresponding siRNA negative control (siControl) or R18 mutant control (*, p < 0.05; n = 3)

Consistent with its known degradative effect on Eag1, Cul7 over-expression resulted in notable reduction of Eag1 protein level in HEK293T cells (Fig. 8A), whereas siRNA knock-down of endogenous Cul7 expression led to more than four-fold increase in Eag1 protein level (Fig. 8B). In the presence of difopein, however, the extent of Cul7-induced Eag1 degradation was significantly diminished (Fig. 8A). This alteration of Eag1 proteostasis appears to be specific for Cul7, as difopein failed to discernibly affect MKRN1-induced degradation of Eag1 **(**Additional file 1: Fig. S6**)**. Upon co-expression with difopein, the potentiation effect of Cul7 knock-down was dramatically decreased (Fig. 8B). The disruption of Cul7-mediated Eag1 degradation by difopein did not seem to involve difopein-induced reduction in Cul7 protein expression, since neither difopein nor 14-3-3 over-expression measurably affected endogenous Cul7 protein level in HEK293T cells **(**Additional file 1: Fig. S7**)**. Importantly, in response to Cul7 knock-down, the regulatory effect of difopein on Eag1 protein level was virtually abolished (Fig. 8B). Overall, these data support the notion that endogenous 14-3-3 proteins may facilitate Eag1 degradation by Cul7.

### 14-3-3 is essential for the interaction between Eag1 and Cul7

**Fig. 9:**
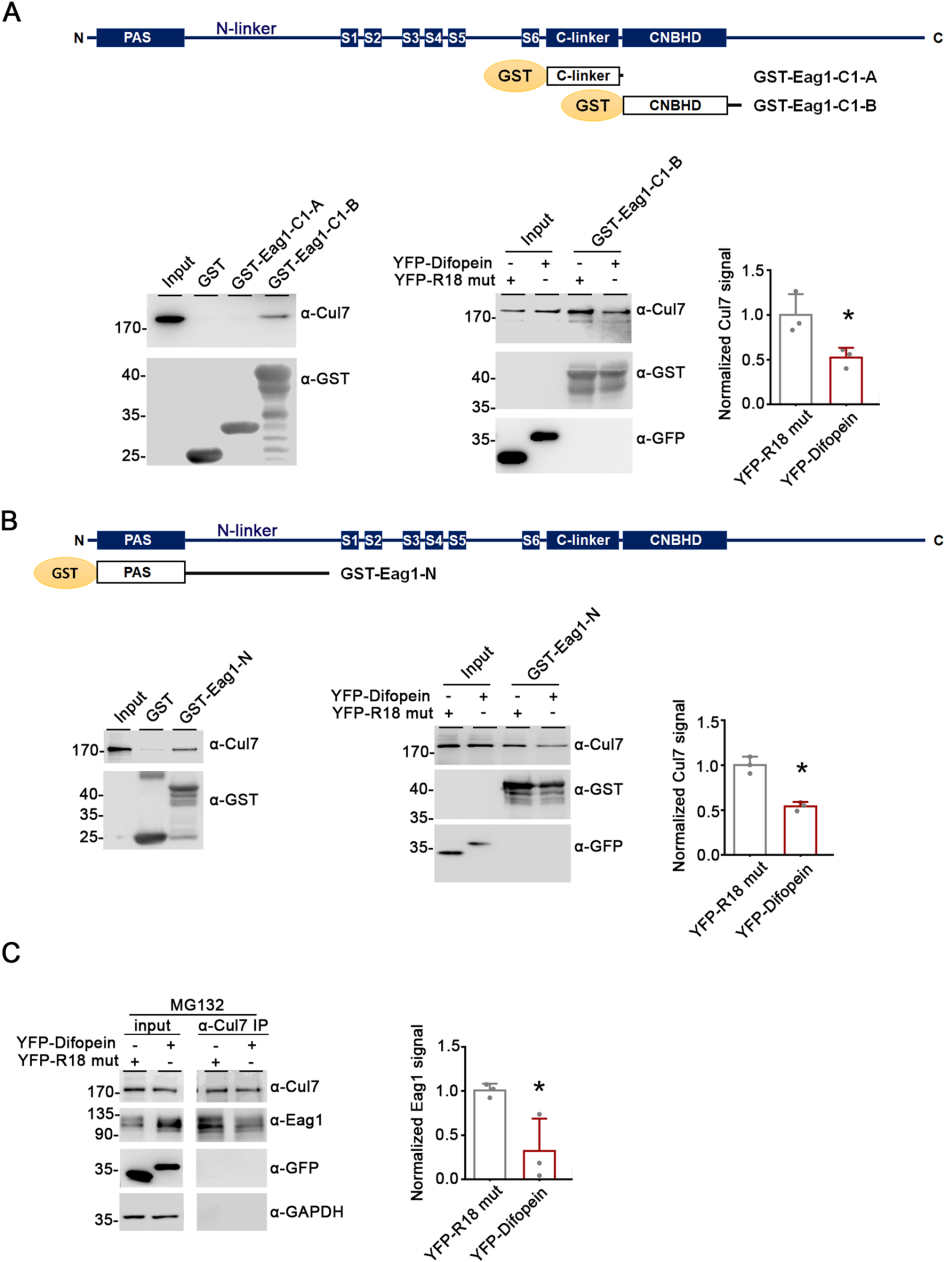
Difopein disrupts Eag1 interaction with Cul7. **A** GST pull-down assay of the interaction of Cul7 with Eag1 C-terminal region. Shown on the top is the structural topology for Eag1, as well as two GST-Eag1 C-terminal fusion proteins, GST-Eag1-C1-A and GST-Eag1-C1-B. (*Left*) Cell lysates prepared from HEK293T cells expressing Myc-Cul7 were used for pull-down assay with GST, GST-Eag1-C1-A, or GST-Eag1-C1-B, followed by immunoblotting with the anti-Cul7 or anti-GST antibodies. (*Center*) Myc-Cul7 was co-expressed with either YFP-R18 mutant or YFP-difopein in HEK293T cells, followed by pull-down assay with GST-Eag1-C1-B. (*Right*) Quantification of the relative pull-down efficiency. Protein densities were standardized as the ratio of Cul7 pull-down signals to the corresponding input signals, followed by normalization with respect to the YFP-R18 mutant co-expression control (*, p < 0.05; n = 3). **B** GST pull-down assay of the interaction of Cul7 with Eag1 N-terminal region. Shown on the top is the structural topology for the N-terminal fusion protein GST-Eag1-N. (*Left*) Cell lysates prepared from HEK293T cells expressing Myc-Cul7 were used for GST pull-down assay with GST or GST-Eag1-N. (*Center*) Myc-Cul7 was pulled down with GST-Eag1-N in the presence of either YFP-R18 mutant or YFP-difopein. (*Rght*) Quantification of the relative pull-down efficiency (*, p < 0.05; n = 3). **C** The effect of difopein on the co-immunoprecipitation efficiency of Cul7 and Eag1 in HEK293T cells. (*Left*) Representative immunoblots. Myc-Cul7, Eag1, and YFP-R18 mutant/YFP-difopein were co-expressed in HEK293T cells. 24 h after transfection, cells were treated with 10 μM MG132 for 12 h. Cell lysates were immunoprecipitated (*IP*) with the anti-Cul7 antibody, followed by immunoblotting analyses. (*Right*) Quantification of relative co-immunoprecipitation efficiency of Cul7 and Eag1. Protein densities were standardized as the ratio of Eag1 IP signals to the corresponding input signals, followed by normalization with respect to the YFP-R18 mutant co-expression control (*, p < 0.05; n = 3)

To ascertain the mechanistic role of 14-3-3 proteins in Cul7-mediated degradation of Eag1, we asked whether inhibition of endogenous 14-3-3 function with difopein may affect the interaction between Eag1 and Cul7. The Eag1 protein contains several well-characterized cytoplasmic structural domains: the Per-Arnt-Sim (PAS) domain (residues 26-136 in rat Eag1) and the adjoining amino (N)-linker (residues 137-214 in rat Eag1) in the N-terminal region, as well as the carboxyl (C)-linker (residues 481-559 in rat Eag1), the cyclic nucleotide-binding homology domain (CNBHD) (residues 560-675 in rat Eag1), and the post-CNBHD segment (residues 676 and beyond in rat Eag1) in the C-terminal region (Fig. 9A) [36, 37]. We first employed the glutathione S-transferase (GST) pull-down assay by using fusion proteins corresponding to three Eag1 structural regions: the C-linker (GST-Eag1-C1-A), the CNBHD and proximal post-CNBHD (GST-Eag1-C1-B), and the PAS domain and N-linker (GST-Eag1-N). The C-terminal CNBHD and proximal post-CNBHD regions of Eag1 are known to play a key role in binding to Cul7 (Fig. 9A) [11]. Remarkably, GST pull-down assays highlighted that the N-terminal region also contributed to the binding of Eag1 to Cul7 (Fig. 9B). Co-expression with difopein resulted in almost 50% reduction in the binding of Cul7 to both the GST-Eag1-C1-B and the GST-Eag1-N fusion proteins (Fig. 9A, B). In line with the GST pull-down results involving Eag1 fusion proteins, immunoprecipitation analyses revealed that binding of full-length Eag1 to Cul7 was substantially decreased by about 70% in response to difopein co-expression (Fig. 9C), indicating that inhibition of endogenous 14-3-3 function with difopein may perturb the interaction between Eag1 and Cul7.

**Fig. 10:**
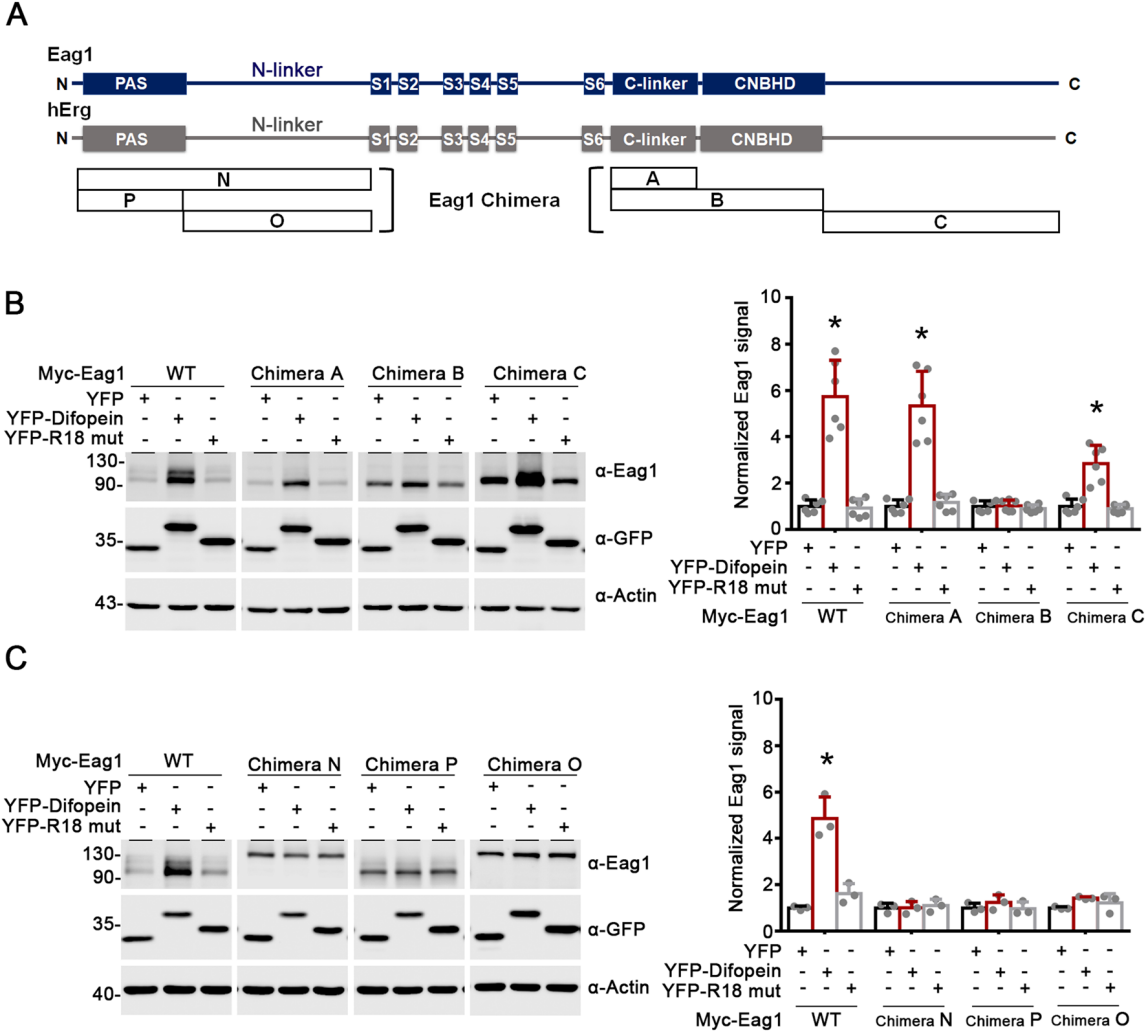
The CNBHD and PAS domain are essential for Eag1 regulation by difopein. **A** Structural topology for Eag1, hErg, and various Eag1 chimeric constructs. Chimera A: Eag1 containing hErg C-linker. Chimera B: Eag1 containing hErg CNBHD. Chimera C: Eag1 containing hErg post-CNBHD region. Chimera N: Eag1 containing the complete hErg N-terminal region. Chimera P: Eag1 containing hErg PAS domain. Chimera O: Eag1 containing hErg N-linker. **B**, **C** Replacement with hErg CNBHD (chimera B), PAS domain (chimeras N and P), or N-linker (chimeras N and O) abolishes the effect of difopein on Eag1 protein level. (*Left panels*) Representative immunoblots. (*Right panels*) Quantification of relative Eag1 protein levels. Myc-tagged Eag1 wild-type (WT) and chimeric constructs were co-transfected with YFP vector, YFP-R18 mutant or YFP-difopein in HEK293T cells. Protein densities were standardized as the ratio of Eag1 signals to the cognate β-actin signals, followed by normalization with respect to the YFP vector control (*, P < 0.05; n = 3–6)

To further understand the structural basis underlying 14-3-3 regulation of Cul7-mediated Eag1 degradation, we employed chimeric constructs of two distinct subtypes of the KCNH channel family: rat Eag1 (Kv10.1; KCNH1) and human Erg (hErg; Kv11.1; KCNH2) (Fig. 10A) [38, 39]. Figure 10B depicts that replacing the CNBHD, but not the C-linker, of Eag1 with that of hErg prevented difopein from boosting Eag1 protein level. Introduction of the post-CNBHD segment of hErg into Eag1 considerably reduced (~ 50%), but did not eliminate, the proteostatic effect difopein (Fig. 10B). Moreover, replacement with the complete N-terminal region, the PAS domain, or the N-linker of hErg resulted in the loss of the up-regulation effect of difopein on Eag1 (Fig. 10C). Together, the foregoing observations are consistent with the idea that the N-terminal PAS domain and N-linker regions, as well as the C-terminal CNBHD and proximal post-CNBHD regions, are essential for the regulation of Eag1-Cul7 interaction by endogenous 14-3-3 proteins.

### 14-3-3 inhibition increases the expression of disease-causing Eag1 mutants

**Fig. 11:**
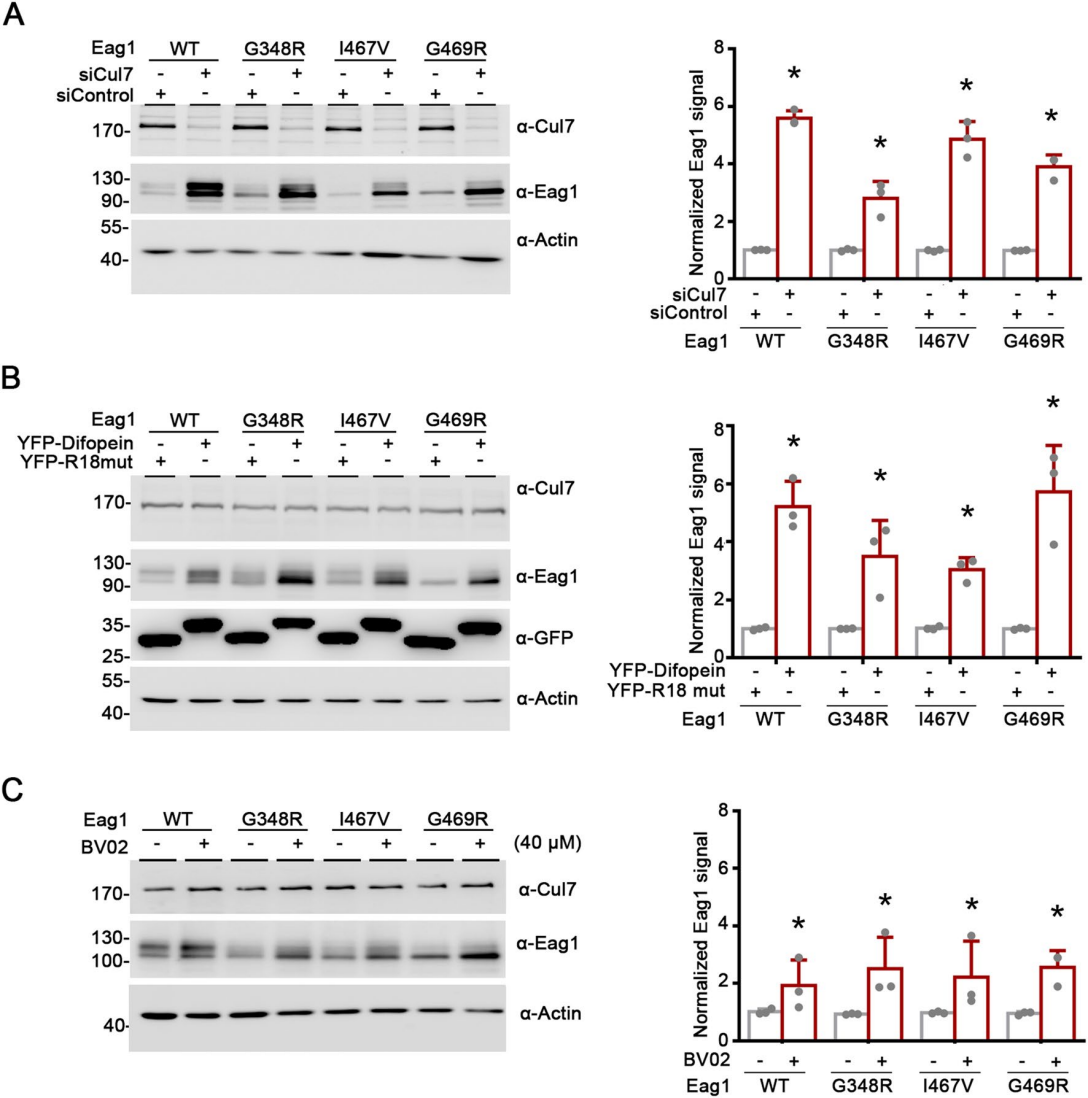
14-3-3 proteins contribute to Cul7-mediated degradation of disease-associated mutant Eag1 proteins. The effect of siRNA knockdown of endogenous Cul7 (**A**), difopein co-expression (**B**), or BV02 treatment (**C**) on WT and mutant Eag1 protein levels in HEK293T cells. (*Left panels*) Representative immunoblots. (*Right panels*) Quantification of relative Eag1 protein levels. Protein densities were standardized as the ratio of Eag1 signals to the cognate β-actin signals, followed by normalization with respect to the YFP-R18 mutant (*, P < 0.05; n = 3)

Mutations associated with the neurodevelopmental disease ZLS manifest functional and proteostatic anomaly of Eag1 K^+^ channel [8–11]. In addition, Cul7 contributes to enhanced protein degradation of the disease-causing Eag1 mutants G348R, I467V, and G469R (Fig. 11A) [11]. We thus asked whether 14-3-3 proteins may also be involved in Cul7-mediated protein degradation of the Eag1 mutants. Figures (11B, C) illustrate that, in HEK293T cells, inhibition of endogenous 14-3-3 function with difopein or BV02 considerably promotes protein expression of the three Eag1 mutants, suggesting that enhanced degradation of ZLS-associated Eag1 mutants may in part be attributable to the modulation of Cul7-Eag1 interaction by 14-3-3 proteins.

## Discussion

### Chaperone-like regulation of Eag1 protein homeostasis by endogenous 14-3-3 proteins

Previous studies from our lab demonstrated functional modulation of Eag1 by its binding partner 14-3-3θ, as well as proteostatic regulation of the K^+^ channel by the E3 ubiquitin ligases Cul7 and MKRN1 [10, 11, 23]. In both neuronal and non-neuronal tissues, 14-3-3 proteins have been shown to regulate substrate protein folding, ubiquitination, and trafficking, consistent with the presence of a significant chaperone-like function [24–27, 40–42]. Furthermore, 14-3-3 proteins may be directly involved in the ubiquitin–proteasome system by promoting the transfer of ubiquitinated substrates to the proteasome [26, 43]. In line with these observations, herein we provided several lines of evidence indicating that endogenous 14-3-3 proteins substantially enhance ubiquitination and degradation of Eag1 by the Cul7 E3 ligase complex, but not by the monomeric MKRN1 E3 ligase. Suppression of endogenous 14-3-3 function with difopein, BV02, or specific RNA interference significantly attenuated the extent of Cul7-mediated Eag1 degradation, and that Cul7 knock-down practically eradicated the effect of difopein on Eag1 protein level. Furthermore, difopein substantially reduced Eag1 protein ubiquitination at both the ER and the plasma membrane. Taken together, we propose that endogenous 14-3-3 proteins promote Cul7-mediated proteasomal and lysosomal degradation of immature and mature Eag1 proteins, respectively.

E3 ubiquitin ligases such as parkin and TRIM25 are subject to 14-3-3-mediated auto-ubiquitination [44, 45]. Therefore, an alternative interpretation of our data is that 14–3-3 proteins may mainly down-regulate protein expression of Cul7, thereby indirectly affecting Eag1 proteostasis. Nonetheless, neither suppressing endogenous 14-3-3 function with difopein nor over-expressing 14-3-3proteins discernibly altered Cul7 protein level **(**Additional file 1: Fig. S7**),** indicating that regulation of Eag1 proteostasis by 14-3-3 does not entail significant change in Cul7 protein expression.

In mammals, 14-3-3 proteins consist of seven isoforms: β, ε, γ, η, τ/θ, ξ, and σ [16–18]. We determined previously that Eag1 binds to 14-3-3 β, γ, η, θ, and ξ isoforms, with β, η, and θ displaying substantially higher binding affinity [23]. By performing shRNA knockdown of specific endogenous 14-3-3 isoforms (β, η, and θ) in both HEK293T cells and neurons, here we noted that Eag1 protein level was effectively modulated by 14-3-3θ and 14-3-3β, but not 14-3-3η. Similar isoform-specific regulation of protein homeostasis has been reported for other 14-3-3 substrate proteins as well. For example, 14-3-3θ promotes protein degradation of the water channel aquaporin-2, whereas 14-3-3ζ prevents aquaporin-2 ubiquitination [46]. Moreover, 14-3-3ε and 14-3-3ζ preferentially attenuate ubiquitin conjugation of the adaptor protein Cdt2 and the cadherin-associated protein β-catenin, respectively [47–49]. Further work will be required to elucidate the precise mechanisms underlying isoform-specific regulation of Eag protein homeostasis by endogenous 14-3-3 proteins.

### Modeling 14-3-3-Eag1-Cul7 interaction

Like other voltage-gated K^+^ channels, a functional Eag1 K^+^ channel is a tetramer, with each subunit containing the N-terminal PAS domain and the C-terminal CNBHD in the intracellular region, as well as six transmembrane helical segments that include the voltage-sensor domain and the ion-conducting pore domain [36]. The PAS domain from a given subunit interacts directly with the CNBHD of a neighboring subunit, known as an intersubunit, domain-swapped arrangement [36]. The primary PAS domain-CNBHD interfaces include a direct interaction of an α-helix (residues 57–61 in rat Eag1) in the PAS domain with an intrinsic ligand motif (YNL; residues 672-674 in rat Eag1) at the distal end of the CNBHD, a salt bridge between the PAS domain (R57 in rat Eag1) and the CNBHD (D615 in rat Eag1), and an interaction of two PAS-domain β strands (residues 29-45 in rat Eag1) with the proximal end (residues 678-687 in rat Eag1) of the post-CNBHD segment [37].

For all subtypes of KCNH channels, including Eag1 and hErg, the direct interaction between the PAS domain and the CNBHD plays a critical role in their voltage-dependent gating functions [50–52]. Moreover, we have previously shown that the PAS domain and CNBHD are essential for the interaction of Eag1 with 14-3-3θ and Cul7 [11, 23] (Fig. 9). Importantly, by employing chimeric constructs between Eag1 and hErg, we demonstrated in the current study that the N-terminal PAS domain and N-linker regions, as well as the C-terminal CNBHD and proximal post-CNBHD regions, are imperative for the proteostatic regulation of Eag1 by 14-3-3 proteins.

**Fig. 12:**
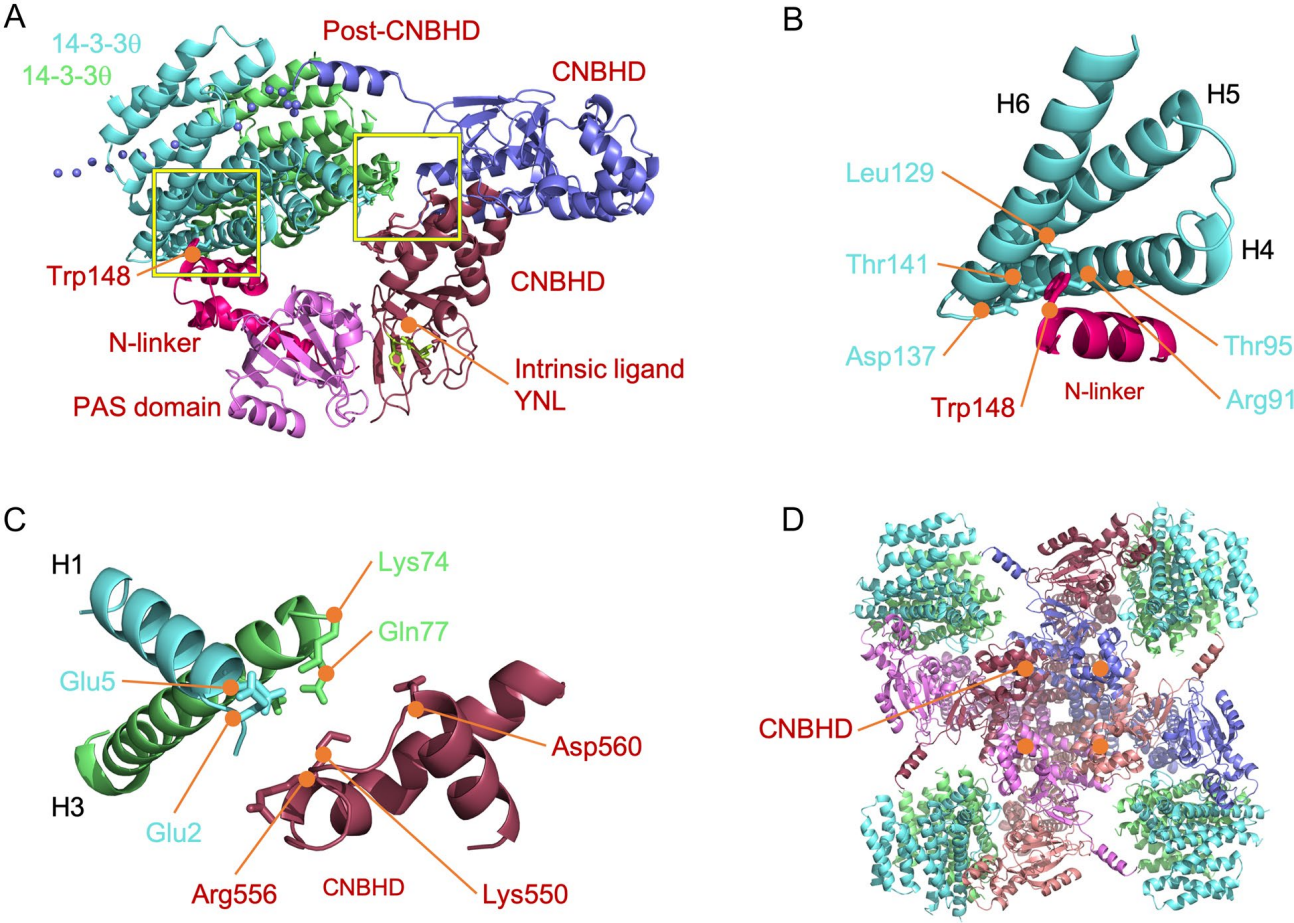
Molecular modeling of the binding of 14-3-3θ homodimer to Eag1. Protein docking models based on the structures of human 14-3-3θ (PDB: 2BTP) and rat Eag1 (PDB: 5K7L). **A** Ribbon representation of a single 14-3-3θ homodimer (colored in *aqua* and *green*) interacting with the N-linker region (*raspberry*) and PAS domain (*violet*) of one Eag1 subunit, the proximal CNBHD (*burgundy*) of a second Eag1 subunit, and the proximal post-CNBHD region (*blue*) of a third Eag1 subunit. The PAS domain (*violet*) from one Eag1 subunit directly interacts with the distal end of the CNBHD (*burgundy*) of a neighboring Eag1 subunit, with the intrinsic ligand motif (YNL) emphasized in *lime*. A portion of the distal segment of the post-CNBHD region (*blue*), which may also be in contact with 14-3-3, is schematically presented as spheres. The two yellow boxes (~ 15 Å × 15 Å) denote the 14-3-3θ-Eag1 binding regions highlighted in (**B**) and (**C**). **B** Enlarged view of the 14-3-3θ-Eag1 binding region enclosed by the yellow box to the left in (**A**), highlighting that the H4, H5, and H6 helices (*aqua*) of the same 14-3-3θ subunit are in close proximity (~ 3–5 Å) with the N-linker (*raspberry*) of Eag1. Specific residues in 14-3-3θ and Eag1 are labeled in *aqua* and *raspberry*, respectively. **C** Enlarged view of the 14-3-3θ-Eag1 binding region enclosed by the yellow box to the right in (**A**), highlighting that the H1 helix (*aqua*) from one 14-3-3θ subunit and the H3 helix (*green*) from the other 14-3-3θ subunit are in close proximity (~ 3–5 Å) with the proximal CNBHD (*burgundy*) of Eag1. Specific residues in the two 14-3-3θ subunits are labeled in *aqua* and *green*, respectively. **D** Intracellular view of four 14-3-3θ homodimers (all in *aqua* and *green*) in contact with the Eag1 tetramer (*violet*, *burgundy*, *blue*, *salmon*). CNBHDs are located in the center region, directly interacting with PAS domains from neighboring Eag1 subunits

**Fig. 13:**
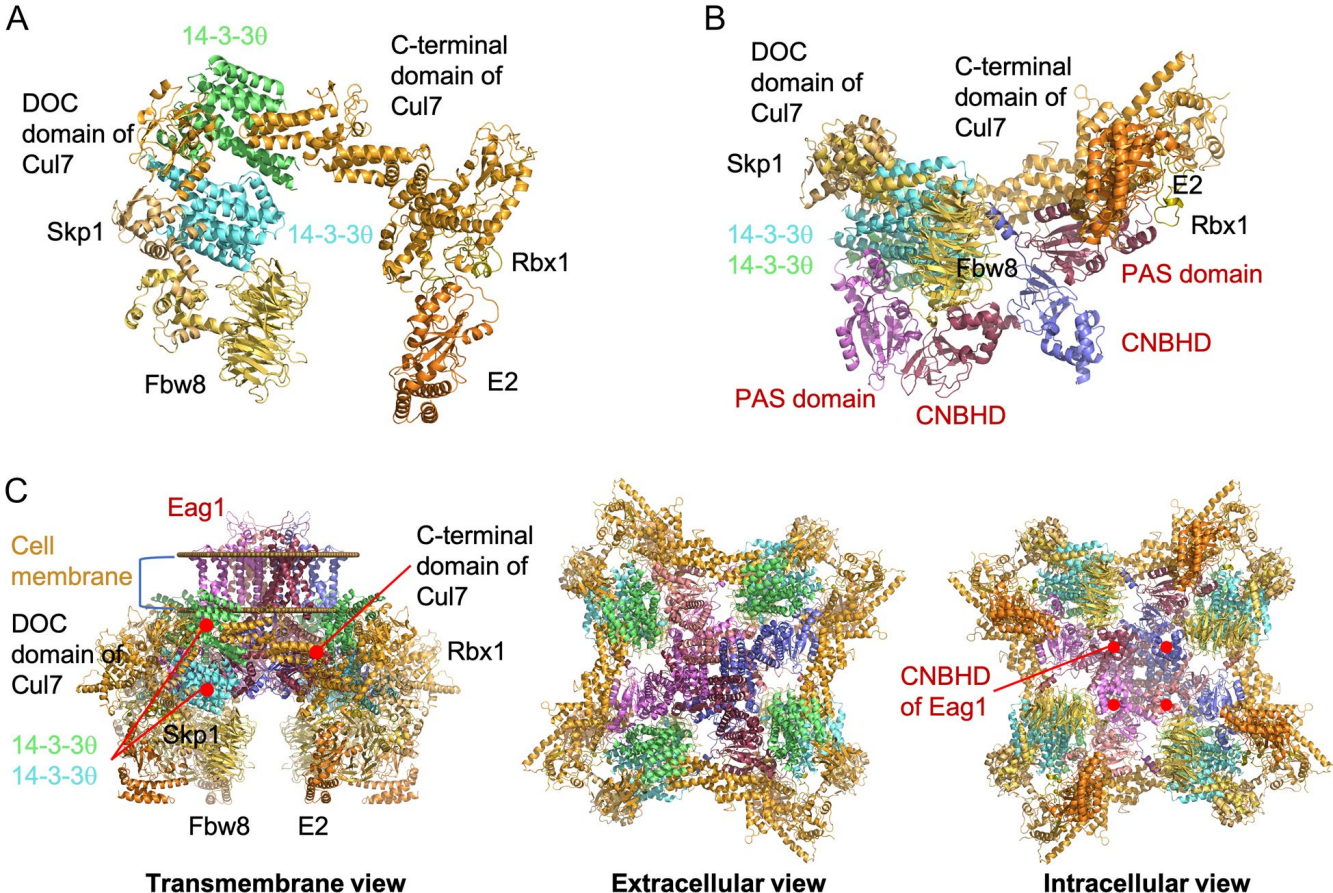
Protein docking models for the 14-3-3-Eag1-Cul7 complex. Homology modeling of the Cul7 protein complex was based on the structures of cullin 1 (PDB: 1LDK), Skp1-Fbw7 complex (PDB: 2OVP), E2 ubiquitin-conjugating enzyme (PDB: 3CEG), Doc1 homology domain (PDB: 1GQP), and CPH domain (PDB: JNG). **A** Interaction of 14-3-3θ homodimer (*aqua*, *green*) with the Cul7 (*tangerine*)-Skp1 (*orange*)-Fbw8 (*saffron*)-Rbx1 (*olive*)-E2 (*pumpkin*) protein complex. One 14-3-3θ subunit (*green*) may directly contact a Cul7 loop region between the DOC domain and C-terminal domain, whereas the other 14-3-3θ subunit (aqua) is modeled as a binding partner of the adaptor protein Skp1. **B** Ternary organization of 14-3-3θ homodimer, Cul7 protein complex, and the PAS domain/CNBHD from three Eag1 subunits (*violet*, *burgundy*, *blue*). The Cul7 complex appears to exclusively bind to a single Eag1 subunit (*burgundy*), with the Cul7 C-terminal domain sitting on the surface of the Eag1 PAS domain, and the substrate-targeting subunit Fbw8 directly contacting the Eag1 CNBHD. As in Fig. 13, 14-3-3θ homodimer (*aqua*, *green*) interacts with the N-linker (*violet*)/CNBHD (*burgundy*)/post-CNBHD (*blue*) from three distinct Eag1 subunits, respectively. Also shown are two sets of intersubunit PAS domain-CNBHD interaction between neighboring Eag1 subunits (*violet*-*burgundy*; *burgundy*-*blue*). **C** Transmembrane, extracellular, and intracellular views of four Cul7 protein complexes, four 14-3-3θ homodimers, and the Eag1 tetramer (*violet*, *burgundy*, *blue*, *salmon*) at the plasma membrane. The docking models in (**A**) and (**B**) are equivalent to the enlargement of a portion of the transmembrane and intracellular views, respectively

14-3-3 proteins function as dimers in cup-like shape, with each subunit consisting of nine antiparallel helices (H1 to H9), as well as an amphipathic ligand binding groove formed by four helices (H3, H5, H7 and H9) [53–55]. To understand the molecular basis of the regulation of Eag1 proteostasis by 14-3-3 proteins, we modeled 14-3-3θ homodimer binding to Eag1 based on the crystal and cryo-electron microscopy (cryo-EM) structures of human 14-3-3θ (PDB: 2BTP) and rat Eag1 (PDB: 5K7L), respectively [36, 56]. As depicted in Fig. 13, our protein docking model supports the idea that 14-3-3θ homodimer interacts with Eag1 in a manner analogous to the well-characterized binding of calmodulin to Eag1 [36, 57]. Each 14-3-3θ homodimer appears to directly interact with the N-linker region close to the PAS domain in one Eag1 subunit, as well as the proximal CNBHD and the proximal post-CNBHD segment from two neighboring Eag1 subunits, respectively (Fig. 12A). In other words, each 14-3-3θ homodimer may simultaneously bind to three subunits of the Eag1 tetramer. Based on our docking model, the H4-H5-H6 helices in one subunit of 14-3-3θ homodimer may clamp an α-helix, including the calmodulin-anchoring residue W148, located in the N-linker region of Eag1 (Fig. 12B). Therefore, rather than serving as 14-3-3 contact region, the PAS domain may indirectly affect the spatial orientation of the N-linker, which could explain why introduction of hErg PAS domain abolished the proteostatic modulation of 14-3-3 proteins on Eag1 chimera P (Fig. 10C). In direct contrast with the interaction of the Eag1 N-terminal region with a single 14-3-3θ subunit of the homodimer, a helix-loop-helix structure at the proximal end of the Eag1 CNBHD seems to be in close proximity with both the H1 helix in one 14-3-3θ subunit and the H3 helix in the other 14-3-3θ subunit (Fig. 12C). Overall, similar to the presence of four calmodulin molecules interacting with an Eag1 tetramer, we propose that four 14-3-3θ homodimers are bound to the Eag1 tetramer (Fig. 12D). Interestingly, by performing sequence alignment of all six 14–3-3 isoforms, we noticed that the amino acid sequence in the abovementioned Eag1 contact regions is not well conserved in 14–3-3 proteins, which may in part contribute to the differential Eag1 proteostatic effects amongst various 14–3-3 isoforms (Fig. 1C, 5C).

We demonstrated here that endogenous Cul7 co-exists in the same protein complex with 14-3-3θ and Eag1 (Additional file 1: Fig. S5). To gain further insight into how 14-3-3 regulates Cul7-Eag1 interaction, we also modeled the interaction of Cul7 with 14-3-3θ and Eag1. In light of the lack of a complete Cul7 protein structure, we performed homology modeling of the Cul7-Skp1-Fbw8-Rbx1-E2 protein complex based on the crystal structures of cullin 1 (PDB: 1LDK), Skp1-Fbw7 complex (PDB: 2OVP), E2 ubiquitin-conjugating enzyme (PDB: 3CEG), and Doc1 homology domain (PDB: 1GQP), as well as the NMR structure of Cul7 CPH domain (PDB: JNG) [58–62], followed by protein docking with 14-3-3θ homodimer and Eag1.

Figure 13A depicts our docking model for the interaction of 14-3-3θ homodimer with a single Cul7 complex. The model suggests that a portion of a ligand-binding pocket of 14-3-3θ may interact with a short linker region between the DOC domain and C-terminal domain of Cul7. In addition, 14-3-3θ may be in direct contact with the adaptor protein Skp1, which recruits the substrate-targeting subunit Fbw8. We therefore speculate that, by directly interacting with Cul7 and Skp1, 14-3-3θ may control the interaction between Fbw8 and substrate protein, thereby modulating the E3 ligase function of the Cul7 protein complex.

Figure 13B further illustrates our docking model for the interaction of the Cul7 E3 ligase complex with Eag1. We propose that the C-terminal domain of Cul7, which binds to the RING-domain protein Rbx1 and thereby recruits the E2 ubiquitin-conjugating enzyme, may sit on the surface of the Eag1 PAS domain. Furthermore, the DOC domain of Cul7, as well as Skp1 and Fbw8, appears to be localized along a cleft between 14-3-3θ and Cul7, such that the substrate recognition protein Fbw8 may bind to the CNBHD of the same Eag1 subunit. In other words, unlike the interaction of 14-3-3θ homodimer with three neighboring Eag1 subunits (Fig. 12), each Cul7 protein complex exclusively binds to a single Eag1 subunit. Therefore, in regard to a functional, tetrameric Eag1 channel located at the plasma membrane, four Cul7 protein complexes may individually interact with distinct Eag1 subunits of the tetramer (Fig. 13C). Taken as a whole, our protein docking models in Figs. 12 and 13 are in agreement with the notion that 14-3-3θ homodimer may regulate Cul7-mediated Eag1 degradation by facilitating the direct interaction between Cul7 E3 ligase complexes and Eag1 subunits.

Previous studies of 14-3-3 oligomerization suggest that both homo- and hetero-dimerization are crucial for 14-3-3 scaffolding function with substrate proteins [55]. It remains to be determined, therefore, whether various 14-3-3 isoforms may regulate Cul7-mediated Eag1 degradation in the form of homodimers or heterodimers. It is also worth mentioning that both phosphorylated and unphosphorylated substrate proteins may be accommodated within the same ligand-binding site of 14-3-3, albeit in opposite orientations [53]. In accordance with our previous demonstration that 14-3-3 proteins seem to regulate Eag1 K^+^ channel activity via a phosphorylation-independent mechanism [23], in the foregoing protein docking models for 14-3-3θ homodimer, Cul7 and Eag1 are presumed to bind to two separate ligand-binding pockets of 14–3-3 dimers in a similar unphosphorylated orientation. The binding of Cul7 and Eag1 to these two separate ligand-binding pockets may induce a change in the conformation of the 14-3-3 dimer interface, which in turn may promote a stable ternary 14-3-3-Eag1-Cul7 complex essential for the downstream proteasomal and lysosomal degradation processes.

### Pathophysiological implication of 14-3-3 regulation of Eag1 proteostasis

Disturbance of protein homeostasis can result in neuronal damage and brain dysfunction [63]. For example, K^+^ channels protect neurons during brain injury by stabilizing membrane potential [64, 65]. Disruption of K^+^ channel proteostasis, therefore, may substantially alter membrane excitability and induce abnormal neuronal action potential firing under pathological conditions [66]. Moreover, brain ischemia may trigger excessive and/or prolonged activation of NMDA receptors, known as excitotoxicity, which instigates a significant state of neuronal death [30, 31, 67]. K^+^ channels are known to play an essential neuroprotective role by counteracting excessive neuronal membrane excitability associated with excitotoxicity [32–34]. Here we demonstrated in cultured neurons that prolonged activation of NMDA receptors led to prominent deterioration of neuronal viability, as well as enhanced proteasomal degradation of Eag1 channels. Furthermore, suppression of endogenous neuronal 14-3-3 function with difopein or BV02 prior to NMDA insult effectively promoted neuronal survival and attenuated NMDA-induced Eag1 protein reduction, implying that preemptive up-regulation of Eag1 protein level via suppressing endogenous 14-3-3 function may effectively protect neurons from NMDA excitotoxicity. Interestingly, 14-3-3γ, a proven Eag1 binding partner, has been implicated in ischemia-induced cell deaths such as ischemic stroke [68–71]. It remains to be determined whether the pathophysiological role of 14-3-3γ in brain ischemia may involve its regulation of Eag1 protein homeostasis.

Unlike its neuron-specific expression pattern in healthy tissues, Eag1 is well known to display ectopic expression in over 70% of human tumors, where over-expression of the K^+^ channel appears to promote cell proliferation and tumor progression [72, 73]. Therefore, Eag1 has been proposed to serve as diagnostic marker, prognostic factor, and therapeutic target in human tumors. Indeed, emerging evidence supports the clinical implication of selective Eag1 inhibitors in cancer treatment [74]. Given the chaperone-like role of 14-3-3 proteins in promoting Cul7-mediated Eag1 degradation, it will be interesting to explore the tumor suppression potential of 14-3-3-targeting chemicals in Eag1-expressing cancers.

Mutations in the genes encoding human Eag1 K^+^ channel have been linked to the congenital neurodevelopmental anomalies TMBTS and ZLS [7–9]. Moreover, previous studies from our lab indicated that ZLS-causing Eag1 mutants are associated with enhanced protein degradation mediated by Cul7 and MKRN1 [10, 11]. We observed in this work that inhibition of endogenous 14-3-3 function with difopein or BV02 noticeably rescued disease-related Eag1 degradation by Cul7, consistent with the idea that 14-3-3 regulation of Eag1 protein homeostasis may play a significant role in the pathophysiology of ZLS. Taken as whole, the foregoing discussions highlight a therapeutic potential of 14-3-3 modulators in correcting disease-causing aberrant Eag1 protein homeostasis.

## Methods

### cDNA constructs

Rat Eag1 cDNA (Dr. Olaf Pongs, Saarland University, Homburg, Germany) was subcloned into the pcDNA3.1 vector (Invitrogen, Carlsbad, CA, USA). Disease-related Eag1 mutations were generated using the QuikChange site-directed mutagenesis kit (Agilent Technologies, Santa Clara, CA, USA), followed by verification with DNA sequencing. cDNAs encoding various 14-3-3 isoforms were isolated from a rat brain cDNA library (OriGene) and subcloned into a modified pcDNA3.1 vector (Invitrogen, Carlsbad, CA, USA) with a myc tag. Yellow fluorescent protein (YFP)-tagged difopein (pSCM138, the 14-3-3-binding antagonist) and the inactive R18 mutant control of difopein (pSCM174) are generous gifts from Dr. Haian Fu (Emory University School of Medicine, Georgia, USA).

### Cell culture and transient transfection

HEK293T cells were maintained in DMEM (Gibco, Gaithersburg, MD, USA) supplemented with 2 mM L-glutamine, 100 units/ml penicillin/streptomycin, and 10% (v/v) fetal bovine serum (HyClone, South Logan, UT, USA). Transient transfection was performed by standard calcium phosphate methods. Two days after transfection, cells were processed for biochemical experiments or immunofluorescence. Where indicated, cycloheximide (Sigma, St. Louis, MO, USA; 100 μg/ml), brefeldin A (LC Laboratories, Woburn, MA, USA; 10 μM), MG132 (Enzo Life Science, Farmingdale, NY, USA; 10 μM), or chloroquine (Sigma, St. Louis, MO, USA; 100 μM) was added to the culture medium.

### Animals and neuronal cultures

All animal procedures were in conformity with the animal protocol approved by the Institutional Animal Care and Use Committee (IACUC) of National Yang Ming Chiao Tung University. Dissociated neuronal cultures were prepared from embryonic embryos (E18 or E19) of Sprague–Dawley rats, using a previously described protocol [75] with a minor modification. In brief, euthanized pregnant rats were dissected for 18-day-old embryos, whose brains were removed and placed in Hank’s balanced salt solution containing 10 mM HEPES (pH 7.4) and 1 mM sodium pyruvate. The cortex and hippocampus were dissected out and dissociated by incubation with 0.25% trypsin solution for 15 min at 37 °C. Fetal bovine serum was added to the above solution to inactivate trypsin activity. Tissue suspension was gently triturated by passing through glass Pasteur pipette for 12 times and filter through a 70 µm strainer into a new 50 ml conical tube. Dissociated neurons were plated onto culture plates coated with 0.2 mg/ml poly-D-lysine (Sigma). Neurons were maintained in the Neurobasal medium (Invitrogen, Carlsbad, CA, USA) supplemented with 2% B27 (Invitrogen, Carlsbad, CA, USA) and 2 mM glutamine in a humidified 5% CO_2_ incubator at 37 °C. Transient transfection of 10 days in vitro (DIV10) neurons was performed by using the Lipofectamine 3000 reagent (Invitrogen, Carlsbad, CA, USA). Various expression constructs were incubated with the transfection reagent for 20 min at room temperature, and DNA-lipofectamine diluted in Opti-MEM (Invitrogen, Carlsbad, CA, USA) was added to culture wells. After 6 h of incubation at 37 °C, the medium was changed and the culture cells were maintained in a 37 °C incubator for 24–48 h.

### Immunoprecipitation and immunoblotting

Cell lysates were prepared by solubilizing cells in the lysis buffer: ice-cold buffer A [(in mM) 100 NaCl, 4 KCl, 2.5 EDTA, 20 NaHCO3, 20 Tris–HCl, pH 7.5, 1 phenylmethylsulfonyl fluoride (PMSF)] with 1% Triton X-100, supplemented with protease inhibitor cocktail (Roche Applied Science, Penzberg, Germany) and PMSF. The lysates were cleared by centrifugation at 12,000 × *g* for 15 min at 4 °C. Protein concentrations were determined by the BCA method (Thermo Scientific, Waltham, MA, USA). For co-immunoprecipitation, solubilized lysates were pre-cleared with protein A/G Sepharose beads (GE Healthcare Biosciences, Piscataway Township, NJ, USA) for 1 h at 4 °C and then incubated for 16 h at 4 °C with protein A/G Sepharose beads pre-coated with desired antibodies. The beads were washed extensively with the buffer A containing 1% Triton X-100, and the retained proteins were analyzed by Western blotting. Protein samples were separated on SDS-PAGE, transferred onto nitrocellulose membrane (GE Healthcare Biosciences, Piscataway Township, NJ, USA), immunoblotted with appropriate dilution of primary antibodies. Blots were then exposed to horseradish-peroxidase-conjugated goat anti-mouse IgG (1:5000), goat anti-rabbit IgG (1:5000), or goat anti-rat IgG (1:5000; Thermo Fisher Scientific, Waltham, MA, USA), and revealed by chemiluminescent detection (Advansta, San Jose, CA, USA). Chemiluminescent signals were then documented and quantified by using GE ImageQuant LAS 4000 detection system and ImageQuant software (GE Healthcare Biosciences, Piscataway Township, NJ, USA). Data shown are representative of at least three independent experiments.

Primary antibodies include rabbit anti-Eag1 (1:15000; Alomone, Jerusalem, Israel), mouse anti-Flag (1:5000; Sigma, St. Louis, MO, USA), rabbit anti-Flag (1:5000; Sigma, Sigma, St. Louis, MO, USA), mouse anti-Cul7 (1:5000; Sigma, St. Louis, MO, USA), mouse anti-ubiquitin (FK2, 1:1000; Enzo Life Sciences, Farmingdale, NY, USA), rabbit anti-GAPDH (1:50000; GeneTex, Irvine, CA, USA), mouse anti-GFP (1:10000; Abcam, Cambridge, MA, USA), mouse anti-β-Actin (1:5000; Sigma, St. Louis, MO, USA), mouse anti-GST (1:5000; Cell signaling Technology, Danvers, MA, USA), and mouse anti-Myc (clone 9E10) antibodies.

### Biotinylation of cell surface proteins

Transfected cells were gently washed with D-PBS [(in mM) 136 NaCl, 2.5 KCl, 1.5 KH_2_PO_4_, 6.5 Na_2_HPO_4_, pH 7.4, 0.9 mM CaCl_2_ and 0.5 mM MgCl_2_), followed by incubation in 1 mg/ml sulfo-NHS-LC-biotin (Thermo Fisher Scientific, Waltham, MA, USA) in D-PBS at 4 °C for 30 min with gentle rocking. To terminate biotinylation, biotin reagents were removed, and cells were rinsed three times with 100 mM glycine in PBS [(in mM) 136 NaCl, 2.5 KCl, 1.5 KH_2_PO_4_, 6.5 Na_2_HPO_4_, pH 7.4], followed by once in TBS buffer [(in mM): 20 Tris–HCl and 150 NaCl, pH 7.4]. Cells were solubilized in the lysis buffer. After centrifugation, insolubilized materials were removed, and solubilized cell lysates were incubated overnight at 4 °C with streptavidin-agarose beads (Thermo Fisher Scientific, Waltham, MA, USA). Beads were washed twice in the lysis buffer, once in a high-salt buffer [(in mM): 500 NaCl, 5 EDTA, 50 Tris–HCl, 0.1% Triton X-100, pH 7.6], followed by once in a low-salt buffer [(in mM): 2 EDTA, 10 Tris–HCl, 0.1% Triton X-100, pH 7.6]. The biotin-streptavidin complexes were eluted from the beads by heating at 70 °C for 5 min in Laemmli sample buffer.

For quantitative analyses, cell lysates from biotinylated intact cells were subject to either direct immunoblotting analyses (*Total*) or streptavidin pull-down prior to immunoblotting (*Surface*). During sample loading for SDS-PAGE, the amount of lysates loaded in the *Total* lane represents about 8% of that used for loading in the *Surface* lane. For both *Total* and *Surface* signals, protein density was standardized as the ratio to the cognate *Total* loading control. Standardized values were normalized with respect to those of the corresponding control group.

### Glutathione S-transferase (GST) pull-down assays

GST fusion proteins were produced and purified by following the manufacturer’s instruction (GE Healthcare Biosciences, Piscataway Township, NJ, USA) as previously reported [11]. In brief, cDNAs encoding specific regions of rat Eag1 were subcloned into the pGEX vector (GE Healthcare Biosciences, Piscataway Township, NJ, USA): GST-Eag1-N (PAS domain and N-linker; amino acids 1-207), GST-Eag1-C1-A (C-linker; amino acids 493-560), and GST-Eag1-C1-B (CNBHD and proximal post-CNBHD; amino acids 561-722). These pGEX-Eag1 constructs were expressed in the *E. coli* strain BL21*.* The lysates of IPTG-induced bacteria were incubated with glutathione-agarose beads (Sigma, St. Louis, MO, USA) that bound GST fusion proteins. GST fusion protein-coated beads (6–8 μg) were consequently incubated overnight with cell lysates prepared from HEK293T cells or dissociated neuronal cultures at 4 ºC, and eluted by heating at 70 ºC for 5 min in the Laemmli sample buffer.

### RNA interference

Transfection of small interference RNAs (siRNAs) (Invitrogen, Carlsbad, CA, USA), including siCul7 #1 (CUL7HSS114814) and negative control (si-Neg: Invitrogen #4390843), was implemented with the Lipofectamine RNAi MAX (Invitrogen, Carlsbad, CA, USA). Cells were plated onto 12-well plates 24 h before siRNA transfection. 24 h after siRNA knockdown, cells were further transfected with Eag1 cDNA. Short hairpin RNAs (shRNAs) in the pLKO.1 puromycin-resistant vector (RNAi Core, Academia Sinica, Taiwan) include sh-14–3-3β#1 (TRCN0000078157), sh-14-3-3β#2 (TRCN0000076400), sh-14-3-3η (TRCN0000222708), sh-14-3-3θ#1 (TRCN0000076393), sh-14-3-3θ#2 (TRCN0000078168), and sh-GFP (TRCN0000207020). For lentiviral packaging in HEK293T cells, cells were transfected with pLKO.1-shRNA, pMD.G and pCMV∆R8.91. Virus-containing medium was harvested and concentrated using ultracentrifugation (10,000 × *g* for 4 h at 4 °C) to yield viral stocks. For lentiviral infection in HEK293T cells, viral stocks were supplemented with 8 μg/ml of polybrene (Sigma, St. Louis, MO, USA). Two days later, infected HEK293T cells were selected by 5 μg/ml of puromycin (Sigma, St. Louis, MO, USA) and subsequently transfected with Eag1 cDNA constructs. For suppressing endogenous 14-3-3 proteins in cultured cortical neurons, DIV10 neurons were infected with viral stocks and subsequently selected with 2 μg/ml of polybrene. After puromycin selection, infected DIV12 neurons were subject to immunoblotting.

### Semi-quantitative reverse transcription polymerase chain reaction (RT-PCR)

Eag1 was co-expressed with various constructs in HEK293T cells. RNA was extracted from HEK293T cells with TRIzol (Sigma-Aldrich), followed by phenol/chloroform separation. RNA was reverse transcribed into cDNA using High-Capacity cDNA Reverse Transcription Kit with RNase Inhibitor (Thermo Scientific). The reaction mixture was incubated at 25 °C and 37 °C for 10 min and 120 min, respectively, followed by heating to 85 °C for 5 min. PCR amplification with Taq polymerase (Thermo Scientific) was performed as follows with SelectCycler II ThermRNA: 94 °C for 3 min; nonsaturating 20 ~ 25 cycles at 95 °C for 30 s, 54 °C for 2 min, and 72 °C for 1 min; and a final extension at 72 °C for 7 min. The specific primers used for PCRs are as follows: Eag1 forward (5ʹ-ATGCCCTCACGGACCATA-3ʹ) and reverse (5’-GCCTGCCTTGGGACCTA-3’); GAPDH forward (5ʹ-AGGGCTGCTTTTAACTCTGGT-3ʹ) and reverse (5ʹ-CCCCACTTGATTTTGGAGGGA-3ʹ); 14-3-3β forward (5ʹ-TGGGCAAAGAGTACCGTGA-3ʹ) and reverse (5ʹ-CCGGATGCAACTTCAGAAAG-3ʹ); 14-3-3η forward (5ʹ-GCGGTGACAGAGCTGAATG-3ʹ) and reverse (5ʹ-TTCAATCTTCTCCCGGTAGG-3ʹ); 14-3-3θ forward (5ʹ-CATTGAGCAGAAGACCGACA-3ʹ) and reverse (5ʹ-CATCGCCACAAGCTACTTCA-3ʹ). The predicted sizes of the amplified PCR products for Eag1, GAPDH, 14-3-3β, 14-3-3η and 14-3-3θ are 173, 207, 172, 186, and 160 base pairs, respectively. RT-PCR products were resolved by electrophoresis in 1.7% agarose gels, followed by quantification with ImageJ. RNA levels of Eag1 were standardized as the ratio of Eag1 signals to the GAPDH mRNA levels, followed by normalization to those of the Myc vector control.

### Immunofluorescence

Cells grown on coverslips were rinsed in ice-cold PBS, and then fixed for 20 min with 4% paraformaldehyde in PBS at 4 °C. After washing with cold PBS, fixed cells were permeabilized and blocked with a blocking buffer (10% normal goat serum in PBS, 0.05% (v/v) Triton X-100) for 60 min at 4 °C. Cells were then incubated with the following primary antibodies overnight at 4 °C: rabbit anti-Eag1 (1:1000; Alomone, Jerusalem, Israel), rabbit anti-calnexin (1:100; Cell signaling Technology, Danvers, MA, USA), or mouse anti-cadherin (1:100; Abcam, Cambridge, MA, USA). After several washes with PBST (PBS with 0.05% Triton X-100), cells were incubated with Alexa Fluor 488-conjugated rabbit IgG and Alexa Fluor 568-conjugated mouse IgG (1:500; Invitrogen, Carlsbad, CA, USA) secondary antibodies for one hour at room temperature. Cell nuclei were labeled with DAPI (1 μg/ml in PBS; Sigma, St. Louis, MO, USA) at room temperature. After final wash, coverslips were mounted in a mounting medium (4% n-propylgallate, 90% glycerol, 0.1 M carbonate buffer, pH 9.2), and viewed using a Zeiss LSM880 laser-scanning microscope (Zeiss, Oberkochen, Germany).

### NMDA excitotoxicity

DIV12 neurons were employed for treatment with NMDA (20 μM; Sigma, St. Louis, MO, USA) for 6 h. Where indicated, prior to NMDA treatment, neurons were pretreated with various inhibitors, including ALLN (10 μM; Enzo, Life Science, Farmingdale, NY, USA), AP5 (50 μM; Sigma, St. Louis, MO, USA), BV02 (40 μM; Sigma, St. Louis, MO, USA), MG132 (20 μM; Enzo, Life Science, Farmingdale, NY, USA), or zVAD-FMK (20 μM; Enzo, Life Science, Farmingdale, NY, USA) in Mg^2+^-free EBSS medium [(in mM) 117 NaCl, 5.3 KCl 1.8 CaCl_2_, 8 MgSO_4_, 1 NaHPO_4_, 26 NaHCO_3_, 5.56 D-Glucose, pH 7.4] for one hour.

### MTT assay

Cell viability was quantitatively evaluated by measuring the capability of mitochondria-dependent reduction of 3-(4,5-Dimethyl-thiazol-2-yl)-2,5-diphenyltetrazolium bromide (MTT). Briefly, cultured neurons were incubated with 12 mM MTT for 4 h under normal culture conditions. The reduction product of tetrazolium salts, the blue-colored formazan, was dissolved in DMSO and quantified photometrically at 540 nm in a scanning microplate reader (TECAN 200 Pro; Tecan Trading AG, Switzerland). The absolute optical density was normalized and expressed as the percentage of cell viability.

### Protein modeling

Protein modeling was performed by using the Phyre2 server [76], which consists of four stages. Protein sequence was first scanned against the specially curated nr20 protein sequence database with HHblits. The resulting multiple-sequence alignment was employed to predict secondary structure with PSIPRED, and both the alignment and secondary structure prediction were combined into a query hidden Markov model. The result of fold library scanning against a database of HMMs of proteins of known structure was used to construct crude backbone-only models. The loop modeling and side-chain placement generated the model candidates. Once a set of models was generated, further evaluation procedures were performed to maximize both confidence and coverage of the query sequence. The final Cα structure was chosen to construct a full backbone using Pulchra, followed by side chain addition using R3.

### Protein–protein docking

The ClusPro web [77] server was selected to perform the prediction of protein–protein docking through three steps: (1) rigid body docking by PIPER sampling billions of conformations, (2) root-mean-square deviation (RMSD) based clustering of the 1000 lowest energy structures generated to find the largest clusters that will represent the most likely models of the complex, and (3) refinement of selected structures using CHARMM energy minimization to obtain the 10 most likely candidates.

### Statistical analyses

Data are presented as mean ± SD. The significance of the difference between two means was tested by Student’s t-test. Means from multiple groups were compared by one-way ANOVA. All statistical analyses and curve fitting were performed with the Origin 7.0 software (Microcal Software, Northampton, MA, USA) or Prism 6 (GraphPad Software, San Diego, CA, USA).

## Supplementary Information

**Additional file 1: Figure S1.** Difopein, but not R18 mutant, interacts with Eag1. Representative immunoblot showing that YFP-difopein, but not YFP-R18 mutant, co-exists in the same protein complex with Myc-14-3-3θ. Myc-14-3-3θ was co-expressed with YFP-difopein or YFP-R18 mutant in HEK293T cells. Immunoprecipitation (IP) was performed with the anti-Myc antibody, followed by immunoblotting with the indicated antibodies. Input volume was 5% of that of the cell lysates for IP. GAPDH was used as the loading control. **Figure S2.** Lack of effect of 14-3-3 over-expression on Eag1 mRNA and protein levels in HEK293T cells. (A-B) Co-expression with Myc-14-3-3β, η, and θ isoforms fails to significantly affect Eag1 mRNA (A) or protein (B) levels in HEK293T cells (n=3-5). (C) Representative immunoblot showing the absence of discernible change in Eag1 protein levels in response to co-expression with the indicated 14-3-3 isoforms in HEK293T cells. **Figure S3.** Quantitative analyses of the immunofluorescent images related to Fig. 4. For a given HEK293T cell, average total Eag1 fluorescence intensity, as outlined by the range of the YFP fluorescence, was measured by using the ImageJ software (National Institutes of Health, Bethesda, MD, USA). Next, the ER and plasma membrane regions within the same cell were outlined in accordance with the staining pattern of calnexin and cadherin, respectively. Average Eag1 fluorescence intensity was then measured in each outlined region, which was then divided by the corresponding total Eag1 fluorescence intensity to determine the relative Eag1 intensity co-localized with calnexin or cadherin. Data were normalized with respect to the corresponding control condition. Each data point represents the mean ratio derived from five to six cells for an independent experiment. Data collected from HEK293T cells over-expressing R18 mutant and difopein are presented as gray and red bars, respectively. Statistical analyses were executed with the Prism software. All numerical data are shown as mean ± standard deviation. Asterisks indicate significant difference from the corresponding control (t-test, P<0.05). **Figure S4.** Quantitative analyses of the immunofluorescent images related to Fig. 5B. The ImageJ software was applied to perform quantitative analyses of endogenous Eag1 protein signals in response to difopein or R18 mutant over-expression in cultured cortical neurons. (Left panel) For a given neuron, average total Eag1 fluorescence intensity within the soma was measured based on the range outlined by the YFP fluorescence. Data were normalized with respect to the corresponding total Eag1 intensity in response to R18 mutant over-expression. Each data point represents the mean intensity derived from three to four neurons for an independent experiment. (Right panel) Eag1 fluorescence puncta number per 100-μm neurite segment was determined by employing the built-in “set scale” and “freehand tool” functions to trace multiple 100-μm neurite segments within a neuron. Each data point represents the mean puncta number derived from three to four neurons (with four neurites per neuron) for an independent experiment. Data collected from neurons over-expressing R18 mutant and difopein are presented as gray and red bars, respectively. Statistical analyses were executed with the Prism software. All numerical data are shown as mean ± standard deviation. Asterisks indicate significant difference from the corresponding control (t-test, P<0.05). **Figure S5.** Interaction of endogenous Cul7 with 14-3-3θ and Eag1 in HEK293T cells and neurons. (A) Co-immunoprecipitation of endogenous Cul7 in HEK293T cells with Myc-14-3-3θ, and Eag1. Myc-14-3-3θ and Eag1 were over-expressed in HEK293T cells. Immunoprecipitation was performed with the anti-Myc antibody, followed by immunoblotting with the indicated antibodies. (B) Interaction of endogenous Cul7 in cultured cortical neurons with GST-14-3-3θ (left) and GST-Eag1-CNBHD (amino acids 561-722; GST-Eag1-C1B) (right) fusion proteins. Cell lysates of cultured cortical neurons were subject to the pull-down assay with the GST-14-3-3θ or GST-Eag1-C1B fusion proteins, followed by immunoblotting with the indicated antibodies. Both endogenous Cul7 and Eag1 proteins were pulled down by GST-14-3-3θ. Likewise, both endogenous Cul7 and 14-3-3θ proteins were pulled down by GST-Eag1-C1B. **Figure S6.** Lack of effect of difopein on MKRN1-induced degradation of Eag1 in HEK293T cells. Eag1 was co-expressed with increasing amounts of Myc-MKRN1 in the presence of YFP-R18 mutant or YFP-difopein in HEK293T cells. (Left) Representative immunoblots. (Right) Quantification of relative Eag1 protein levels with respect to the amount of MKRN1 used for co-transfection (n=3). **Figure S7.** Lack of effect of difopein or 14-3-3 co-expression on endogenous Cul7 level in HEK293T cells. Over-expression of YFP-R18 mutant or YFP-difopein (A), as well as various Myc-14-3-3 proteins (14-3-3β, 14-3-3η, and 14-3-3θ) (B), in HEK293T cells. (Left panels) Representative immunoblots. (Right panels) Quantification of relative endogenous Cul7 protein levels (n=3-6).

## Data Availability

The plasmids and datasets generated during and/or analyzed during the current study are available from the corresponding authors on reasonable request.

## Abbreviations

BFA: Brefeldin A
C: Carboxyl
CHX: Cycloheximide
CNBHD: Cyclic nucleotide-binding homology domain
CQ: Chloroquine
cryo-EM: Cryo-electron microscopy
Cul7: Cullin 7
DIV: Day in vitro
Eag: Ether-à-go-go
ER: Endoplasmic reticulum
GST: Glutathione S-transferase
HEK: Human embryonic kidney
K^+^: Potassium
MKRN1: Makorin ring finger protein 1
N: Amino
NMDA: N-methyl-D-aspartate
PAS: Per-Arnt-Sim
PMSF: Phenylmethylsulfonyl fluoride
RING: Really interesting
shRNA: Short hairpin RNA
siRNA: Small interference RNA
RT-PCR: Reverse transcription polymerase chain reaction
TMBTS: Temple–baraitser syndrome
Ub: Ubiquitin
WT: Wild-type
YFP: Yellow fluorescent protein
ZLS: Zimmermann–laband syndrome
