# Supplementary material for: 14-3-3 proteins regulate cullin 7-mediated Eag1 degradation
Source: Cell Biosci. 2023 Jan 30;13:18. doi: 10.1186/s13578-023-00969-w (PMC9885684; doi:10.1186/s13578-023-00969-w)

**14-3-3 proteins regulate cullin 7-mediated Eag1 degradation**

*Cell & Bioscience*

Chang-Heng Hsieh^1^, Chia-Cheng Chou^2^, Ya-Ching Fang^1,3^ , Po-Hao Hsu^1,3^, Yi-Hung Chiu^1^, Chi-Sheng Yang^1^, Guey-Mei Jow^4^, Chih-Yung Tang^3*^, and Chung-Jiuan Jeng^1,5*^

^1^Institute of Anatomy and Cell Biology, College of Medicine, National Yang Ming Chiao Tung University, Taipei, Taiwan; ^2^National Laboratory Animal Center, National Applied Research Laboratories, Taipei, Taiwan; ^3^Department of Physiology, College of Medicine, National Taiwan University, Taipei, Taiwan; ^4^School of Medicine, Fu-Jen Catholic University, New Taipei City, Taiwan; ^5^Brain Research Center, National Yang Ming Chiao Tung University, Taipei, Taiwan

****Corresponding authors:***

Dr. Chung-Jiuan Jeng

Institute of Anatomy and Cell Biology, College of Medicine, National Yang Ming Chiao Tung University, Taipei 112, Taiwan

Phone: 886-2-28267072

Fax: 886-2-28212884

*E-mail address:* cjjeng@nycu.edu.tw

Dr. Chih-Yung Tang

Department of Physiology, College of Medicine, National Taiwan University, Taipei 100, Taiwan.

Phone: 886-2-23562215.

Fax: 886-2-23964350.

*E-mail address:* tang@ntu.edu.tw

**Additional Information**

Additional file Figures S1-S7.

**Additional file Figure S1. Difopein, but not R18 mutant, interacts with Eag1.**

Representative immunoblot showing that YFP-difopein, but not YFP-R18 mutant, co-exists in the same protein complex with Myc-14-3-3θ. Myc-14-3-3θ was co-expressed with YFP-difopein or YFP-R18 mutant in HEK293T cells. Immunoprecipitation (IP) was performed with the anti-Myc antibody, followed by immunoblotting with the indicated antibodies. Input volume was 5% of that of the cell lysates for IP. GAPDH was used as the loading control.


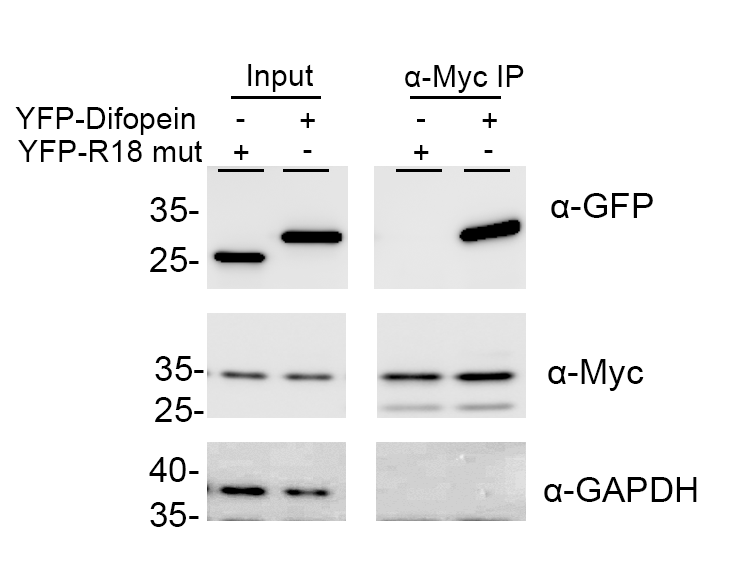


**Additional file Figure S2. Lack of effect of 14-3-3 over-expression on Eag1 mRNA and protein levels in HEK293T cells.**

(A-B) Co-expression with Myc-14-3-3β, η, and θ isoforms fails to significantly affect Eag1 mRNA (A) or protein (B) levels in HEK293T cells (n=3-5). (C) Representative immunoblot showing the absence of discernible change in Eag1 protein levels in response to co-expression with the indicated 14-3-3 isoforms in HEK293T cells.


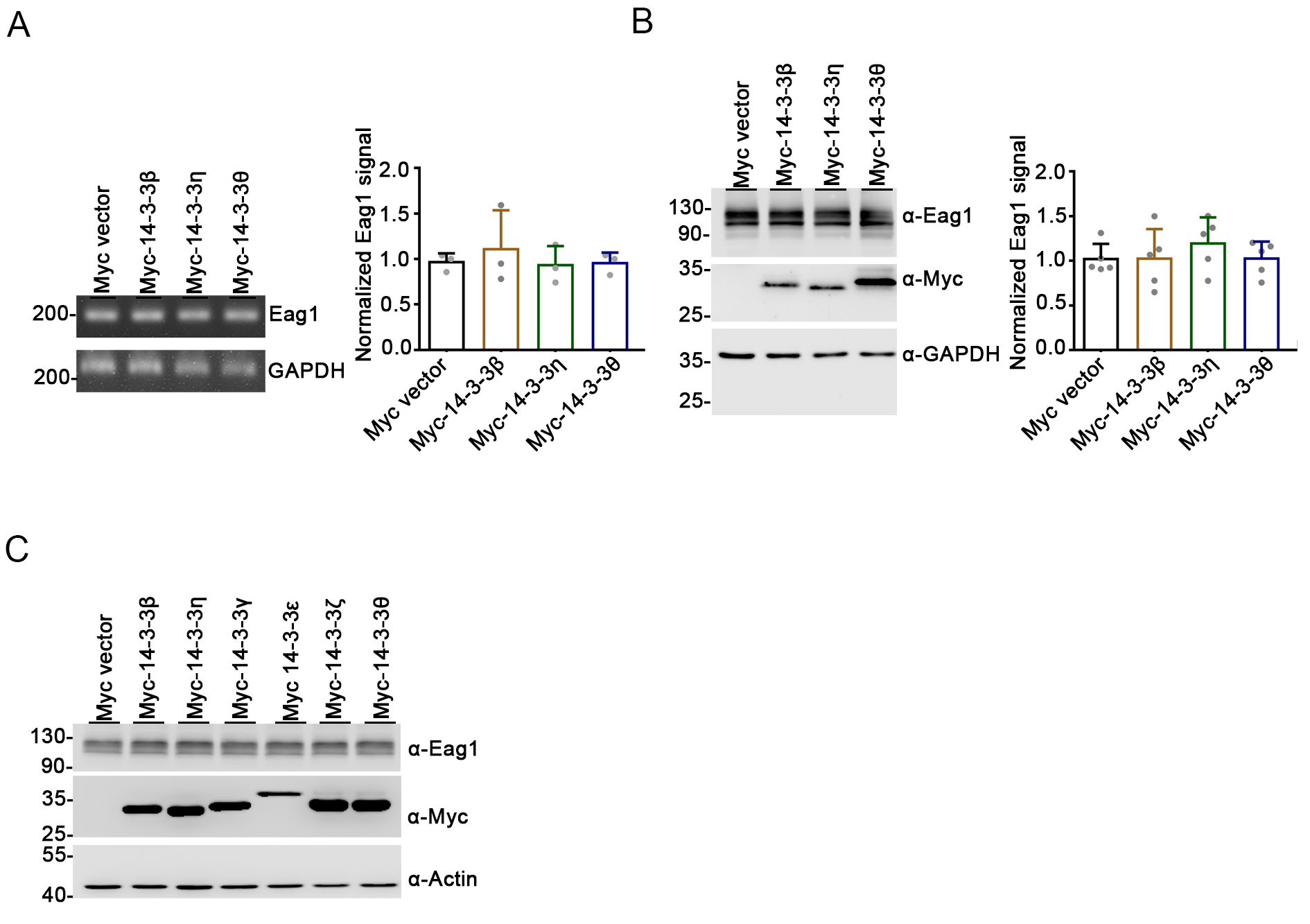


**Additional file Figure S3. Quantitative analyses of the immunofluorescent images related to Figure 4.**

For a given HEK293T cell, average total Eag1 fluorescence intensity, as outlined by the range of the YFP fluorescence, was measured by using the ImageJ software (National Institutes of Health, Bethesda, MD, USA). Next, the ER and plasma membrane regions within the same cell were outlined in accordance with the staining pattern of calnexin and cadherin, respectively. Average Eag1 fluorescence intensity was then measured in each outlined region, which was then divided by the corresponding total Eag1 fluorescence intensity to determine the relative Eag1 intensity co-localized with calnexin or cadherin**.** Data were normalized with respect to the corresponding control condition. Each data point represents the mean ratio derived from five to six cells for an independent experiment. Data collected from HEK293T cells over-expressing R18 mutant and difopein are presented as gray and red bars, respectively. Statistical analyses were executed with the Prism software. All numerical data are shown as mean ± standard deviation. Asterisks indicate significant difference from the corresponding control (t-test, P<0.05).


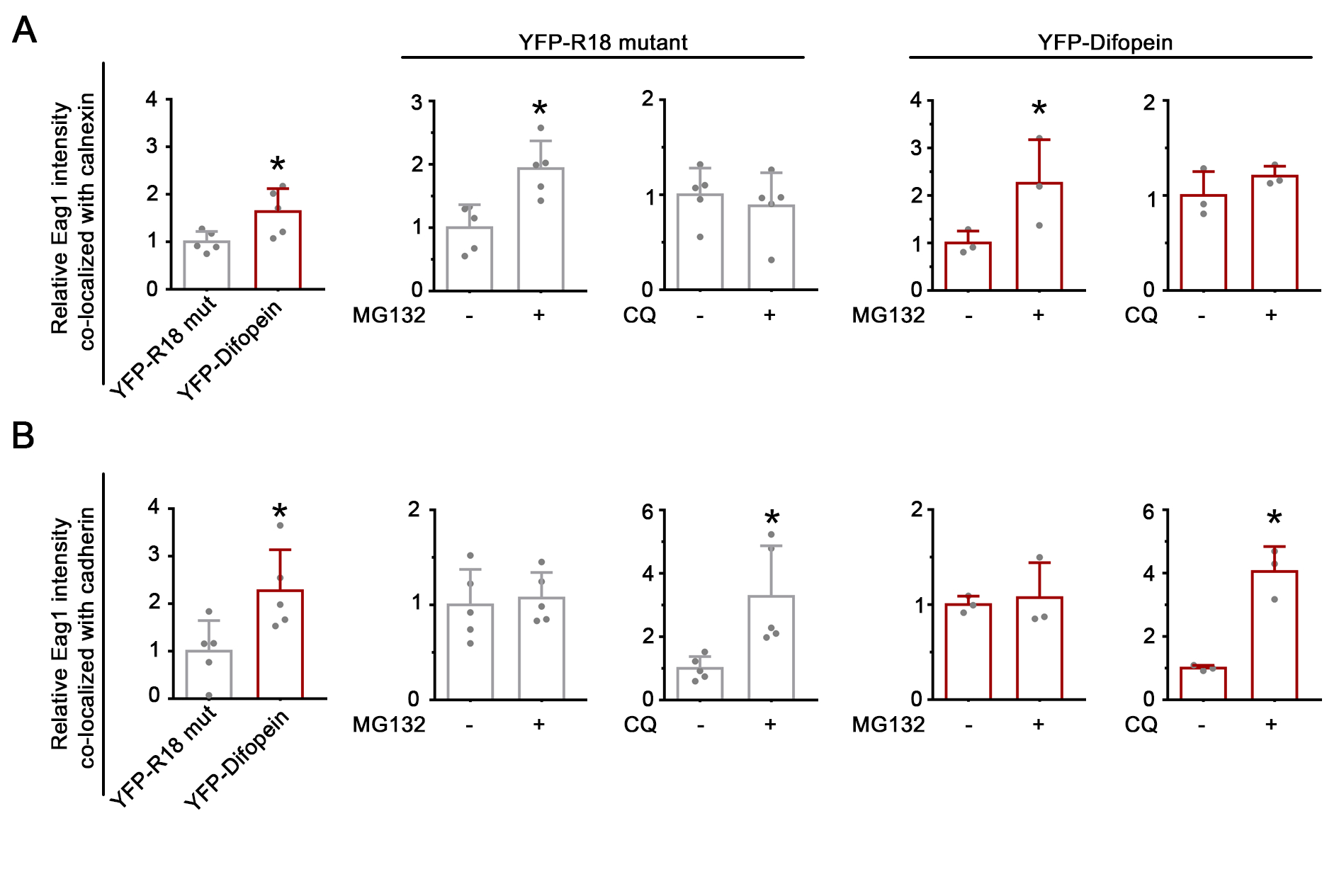


**Additional file Figure S4. Quantitative analyses of the immunofluorescent images related to Figure 5B.**

The ImageJ software was applied to perform quantitative analyses of endogenous Eag1 protein signals in response to difopein or R18 mutant over-expression in cultured cortical neurons. (*Left panel*) For a given neuron, average total Eag1 fluorescence intensity within the soma was measured based on the range outlined by the YFP fluorescence. Data were normalized with respect to the corresponding total Eag1 intensity in response to R18 mutant over-expression. Each data point represents the mean intensity derived from three to four neurons for an independent experiment. (*Right panel*) Eag1 fluorescence puncta number per 100-μm neurite segment was determined by employing the built-in “set scale” and “freehand tool” functions to trace multiple 100-μm neurite segments within a neuron. Each data point represents the mean puncta number derived from three to four neurons (with four neurites per neuron) for an independent experiment. Data collected from neurons over-expressing R18 mutant and difopein are presented as gray and red bars, respectively. Statistical analyses were executed with the Prism software. All numerical data are shown as mean ± standard deviation. Asterisks indicate significant difference from the corresponding control (t-test, P<0.05).


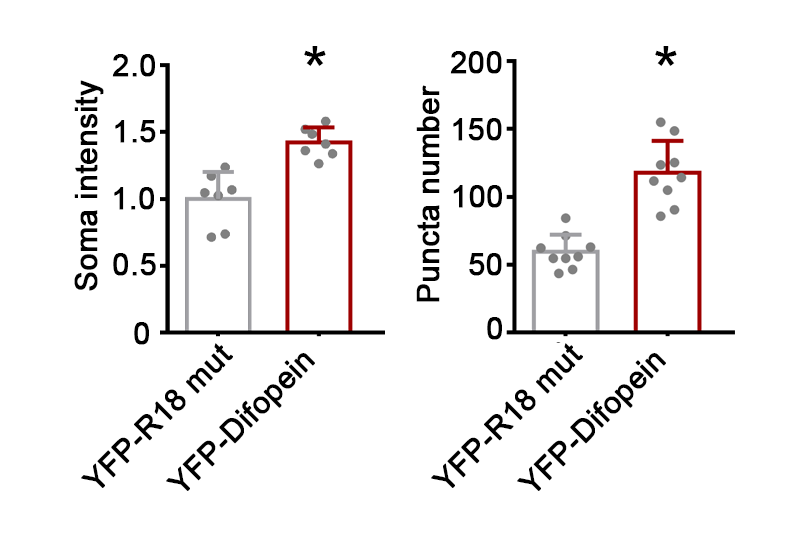


**Additional file Figure S5. Interaction of endogenous Cul7 with 14-3-3θ and Eag1 in HEK293T cells and neurons.**

(A) Co-immunoprecipitation of endogenous Cul7 in HEK293T cells with Myc-14-3-3θ, and Eag1. Myc-14-3-3θ and Eag1 were over-expressed in HEK293T cells. Immunoprecipitation was performed with the anti-Myc antibody, followed by immunoblotting with the indicated antibodies. (B) Interaction of endogenous Cul7 in cultured cortical neurons with GST-14-3-3θ (*left*) and GST-Eag1-CNBHD (amino acids 561-722; GST-Eag1-C1B) (*right*) fusion proteins. Cell lysates of cultured cortical neurons were subject to the pull-down assay with the GST-14-3-3θ or GST-Eag1-C1B fusion proteins, followed by immunoblotting with the indicated antibodies. Both endogenous Cul7 and Eag1 proteins were pulled down by GST-14-3-3θ. Likewise, both endogenous Cul7 and 14-3-3θ proteins were pulled down by GST-Eag1-C1B.


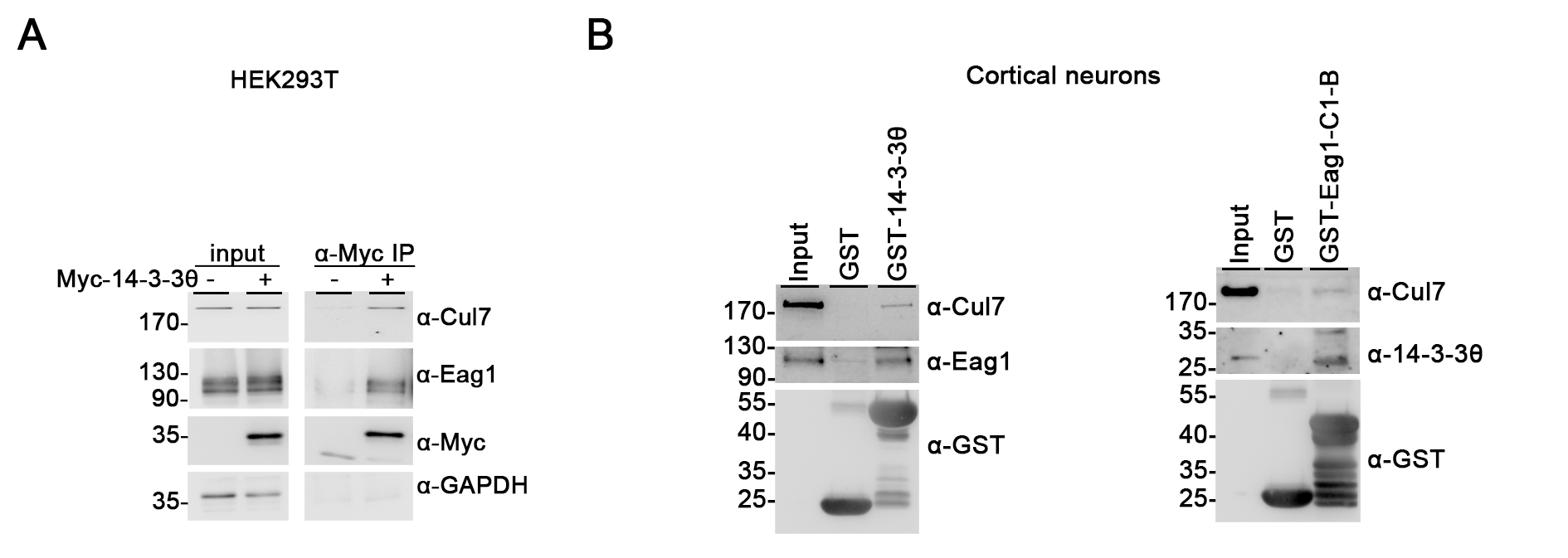


**Additional file Figure S6. Lack of effect of difopein on MKRN1-induced degradation of Eag1 in HEK293T cells.**

Eag1 was co-expressed with increasing amounts of Myc-MKRN1 in the presence of YFP-R18 mutant or YFP-difopein in HEK293T cells. *(Left*) Representative immunoblots. (*Right*) Quantification of relative Eag1 protein levels with respect to the amount of MKRN1 used for co-transfection (n=3).


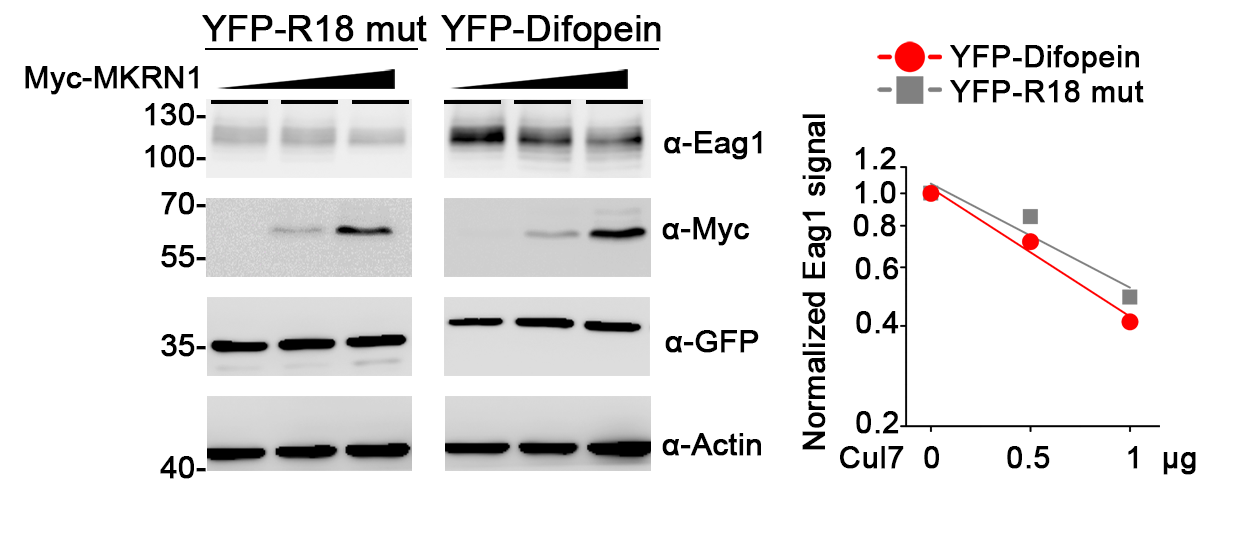


**Additional file Figure S7. Lack of effect of difopein or 14-3-3 co-expression on endogenous Cul7 level in HEK293T cells**

Over-expression of YFP-R18 mutant or YFP-difopein (A), as well as various Myc-14-3-3 proteins (14-3-3β, 14-3-3η, and 14-3-3θ) (B), in HEK293T cells. (*Left panels*) Representative immunoblots. (*Right panels*) Quantification of relative endogenous Cul7 protein levels (n=3-6).


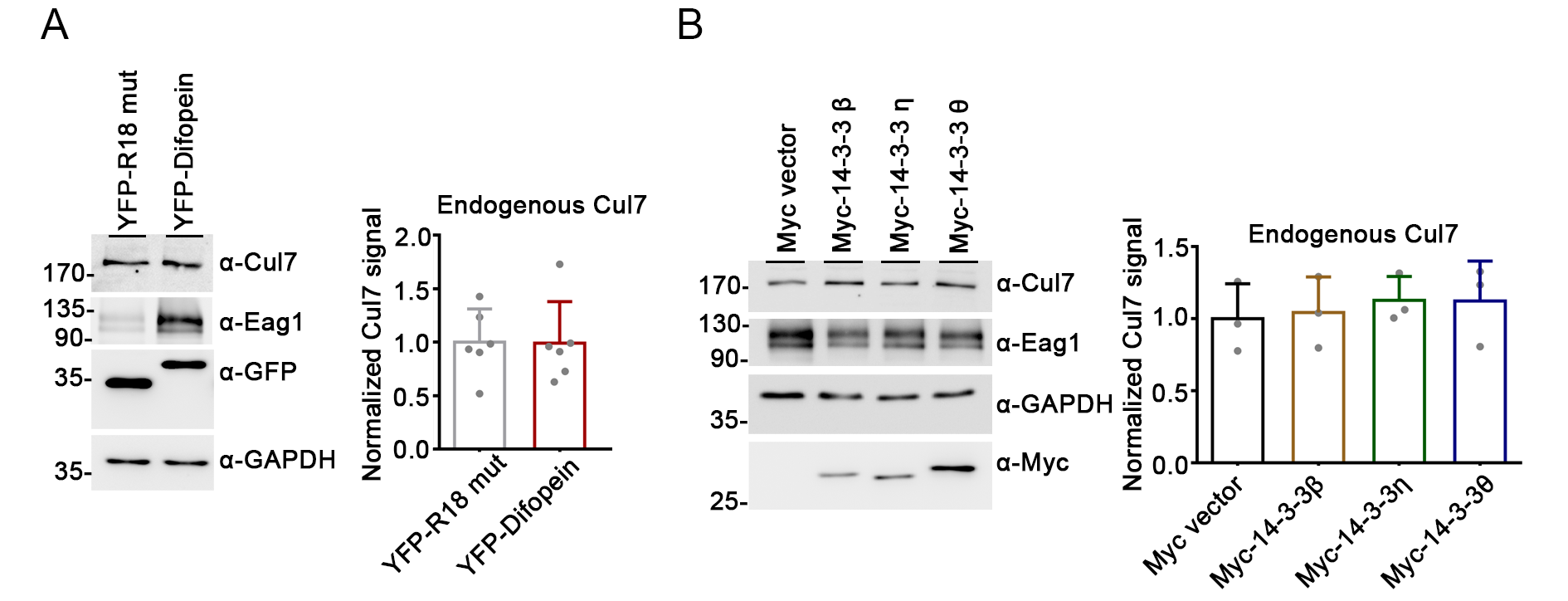

Supplement: Supplementary file 1 — Additional file 1: Figure S1. Difopein, but not R18 mutant, interacts with Eag1. Representative immunoblot showing that YFP-difopein, but not YFP-R18 mutant, co-exists in the same protein complex with Myc-14-3-3θ. Myc-14-3-3θ was co-expressed with YFP-difopein or YFP-R18 mutant in HEK293T cells. Immunoprecipitation (IP) was performed with the anti-Myc antibody, followed by immunoblotting with the indicated antibodies. Input volume was 5% of that of the cell lysates for IP. GAPDH was used as the loading control. Figure S2. Lack of effect of 14-3-3 over-expression on Eag1 mRNA and protein levels in HEK293T cells. (A-B) Co-expression with Myc-14-3-3β, η, and θ isoforms fails to significantly affect Eag1 mRNA (A) or protein (B) levels in HEK293T cells (n=3-5). (C) Representative immunoblot showing the absence of discernible change in Eag1 protein levels in response to co-expression with the indicated 14-3-3 isoforms in HEK293T cells. Figure S3. Quantitative analyses of the immunofluorescent images related to Fig. 4. For a given HEK293T cell, average total Eag1 fluorescence intensity, as outlined by the range of the YFP fluorescence, was measured by using the ImageJ software (National Institutes of Health, Bethesda, MD, USA). Next, the ER and plasma membrane regions within the same cell were outlined in accordance with the staining pattern of calnexin and cadherin, respectively. Average Eag1 fluorescence intensity was then measured in each outlined region, which was then divided by the corresponding total Eag1 fluorescence intensity to determine the relative Eag1 intensity co-localized with calnexin or cadherin. Data were normalized with respect to the corresponding control condition. Each data point represents the mean ratio derived from five to six cells for an independent experiment. Data collected from HEK293T cells over-expressing R18 mutant and difopein are presented as gray and red bars, respectively. Statistical analyses were executed with the Prism software. All [file 13578_2023_969_MOESM1_ESM.docx]
